# Traditional uses, phytochemistry, pharmacology, quality control and clinical studies of Cimicifugae Rhizoma: a comprehensive review

**DOI:** 10.1186/s13020-024-00937-7

**Published:** 2024-05-07

**Authors:** Qianqian Zhang, Wei Wei, Xingyue Jin, Jin Lu, Shujing Chen, Omachi Daniel Ogaji, Shaoxia Wang, Kunze Du, Yanxu Chang, Jin Li

**Affiliations:** 1 State Key Laboratory of Component-based Chinese Medicine, Tianjin University of Traditional Chinese Medicine, Tianjin, 301617 China; 2 Tianjin Key Laboratory of Phytochemistry and Pharmaceutical Analysis, Tianjin University of Traditional Chinese Medicine, Tianjin, 301617 China; 3 Haihe Laboratory of Modern Chinese Medicine, Tianjin, 301617 China

**Keywords:** Cimicifugae Rhizoma, Phytochemistry, Triterpenoid saponins, Pharmacology, Quality control

## Abstract

Cimicifugae Rhizoma, generally known as “Sheng Ma” in China, has great medicinal and dietary values. Cimicifugae Rhizoma is the dried rhizome of *Cimicifuga foetida* L., *Cimicifuga dahurica* (Turcz.) Maxim. and *Cimicifuga heracleifolia* Kom., which has been used to treat wind-heat headache, tooth pain, aphtha, sore throat, prolapse of anus and uterine prolapse in traditional Chinese medicine. This review systematically presents the traditional uses, phytochemistry, pharmacology, clinical studies, quality control and toxicity of Cimicifugae Rhizoma in order to propose scientific evidence for its rational utilization and product development. Herein, 348 compounds isolated or identified from the herb are summarized in this review, mainly including triterpenoid saponins, phenylpropanoids, chromones, alkaloids, terpenoids and flavonoids. The crude extracts and its constituents had various pharmacological properties such as anti-inflammatory, antitumor, antiviral, antioxidant, neuroprotective, anti-osteoporosis and relieving menopausal symptoms. The recent research progress of Cimicifugae Rhizoma in ethnopharmacology, phytochemistry and pharmacological effects demonstrates the effectiveness of its utilization and supplies valuable guidance for further research. This review will provide a basis for the future development and utilization of Cimicifugae Rhizoma.

**Supplementary Information:**

The online version contains supplementary material available at 10.1186/s13020-024-00937-7.

## Introduction

Cimicifugae Rhizoma belonging to the Ranunculaceae, is widely distributed in China, Korea Peninsula, Mongolia and Russia Far East [1]. It was initially recorded in *Shennong’s Classic of Materia Medica* with the functions of detoxification, dispelling warm diseases, preventing pathogen and was thought to be the top grade [2]. According to the 2020 Edition of Chinese Pharmacopoeia (ChP), Cimicifugae Rhizoma is pungent, slightly sweet and cold, belonging in the lung, spleen, stomach and large intestine meridians [3]. It has the effect of effusing the external to outthrust rashes, heat-clearing and detoxifying and Yang-Invigorating. Therefore, Cimicifugae Rhizoma is extensively applied to relieve wind and heat headache, toothache, sore mouth and throat, uterine prolapse, prolapse of anus, etc. [3]. Additionally, it was taken as soil pesticides to eliminate potato tuber moth, fly maggots [4]. In China, Cimicifugae Rhizoma is stipulated as a food supplement and the fresh products have the efficacy of detoxification, detumescence and relieving throat disorder [5]. In early spring, tender leaves can be consumed as cold dishes, stir fried dishes and the filling for staple foods with high edible value [6]. At present, there are nearly 80 kinds of traditional Chinese patent medicines available in the market containing Cimicifugae Rhizoma. Among them, Shengma Pills, Shengma Biejia Pills, Ximingting Pills, Shengti Capsules are in high demand [7, 8]. Owing to the good medicinal properties of Cimicifugae Rhizoma, the application for this herb has gradually increased in recent decades [9, 10].

In China, the dried rhizomes of the three *Cimicifuga* species, *Cimicifuga foetida* L., *Cimicifuga dahurica* (Turcz.) Maxim. and *Cimicifuga heracleifolia* Kom., are referred as the official sources of Cimicifugae Rhizoma [3]. Except the above cultivars, *Actaea purpurea* [11], *Actaea japonica*, *Cimicifuga simplex* [12] black cohosh [13] and *Actaea asiatica Hara* [14] are often used as counterfeits or fake products regionally due to its similarity in the plant appearance and efficacy. Although the application of these products may be beneficial for obtaining materials locally and addressing resource shortages. It has led to unclear varieties and uneven quality of medicinal herbs on the market, which may affect their clinical efficacy and safety. It is necessary to strengthen the quality control of traditional Chinese medicines (TCMs) in order to meet the needs of high-quality medicinal materials circulating in the market and ensure the effectiveness and safety of clinical medications [15].

Recently, an increasing number of modern studies have concentrated on the phytochemical and pharmacological interests of Cimicifugae Rhizoma due to the extensive application. The main chemical constituents of Cimicifugae Rhizoma are triterpenoid saponins (mostly 9,19-cycloartane type), phenylpropanoids, chromones, alkaloids and terpenoids [16]. The crude extracts and monomers of Cimicifugae Rhizoma exhibit extensive pharmacological activity, including anti-inflammatory, antitumor, antiviral, anti-osteoporosis, antioxidant, neuroprotective, angiogenesis and analgesic activities [1]. Moreover, the therapeutic efficacy of Cimicifugae Rhizoma was increased when it was compatible with other herbal medicine, such as *Atractylodes lancea* (Thunb.) DC., *Pueraria lobata* (Willd.) Ohwi [17]. Due to its definite clinical efficacy, Cimicifugae Rhizoma has gained widespread attention in phytochemistry and pharmacology.

Some researchers have systematically reviewed the traditional uses, phytochemistry and pharmacological effects of the *Cimicifuga* genus before 2017 with a relatively high proportion of *Cimicifuga racemose* [1]. Due to its high efficiency and low toxicity, research on Cimicifugae Rhizoma has been continuously increasing in recent years. Moreover, the latest progress in new compounds identification, pharmacological activity and quality control of Cimicifugae Rhizoma have not been reviewed. Therefore, this article intend to systematically review the botanical characterization, chemical composition, pharmacological action, the traditional uses, quality control, clinical study together with toxicity of the official Cimicifugae Rhizoma species in ChP, which provides a good reference for the subsequent development of Cimicifugae Rhizoma and related products in the future.

## Materials and methods

Information related to Cimicifugae Rhizoma on literatures was collected from PubMed, Science Direct, Baidu Scholar, Google Scholar and the China National Knowledge Infrastructure (CNKI) from 1993 to 2023. The following keywords were retrieved from these websites, including “Cimicifugae Rhizoma”, “*Cimicifuga*”, “*C. heracleifolia*”, “*C. dahurica*”, “*C. foetida*”, “cohosh”, “ethnopharmacological use”, “triterpenoid saponins”, “phenylpropanoids”.

## Ethnobotany

### Botanical features and distribution

The *Cimicifuga* plant is a perennial herb. The surface of the root is generally black and cross-section owns many invaginated and round hole-shaped old stems [18]. The stem of *Cimicifuga* plant is 1–2 m high with branching shape and its base is about 1.4 cm. There are nearly polypinnate compound leaves, rhombic in shape, serrated in edge and stalked in short. Flowers are bisexual, bracts are subulate, shorter than pedicels. Seeds are oval and brown with 2.5–3 mm long. However, there are obvious differences in appearances among the official *Cimicifuga*. The details were showed at Table 1.

**Table 1:** Analysis of differences in appearance of the *Cimicifuga* plant

Plant organ	*C. foetida*	*C. heracleifolia*	*C. dahurica*	References
Stem	Base pubescent	Base glabrous	The base is glabrous or slightly hairy	[18]
Flowers	Inflorescence has 3–20 branches Carpels are 2–5, with sessile or very short stalks, densely covered with gray hairs	Inflorescence has 2–9 branches Carpels are 3–5, short stalked and glabrous	Inflorescence are more than 7–20 branches Carpels are 4–7, with sessile or shortly stalked, sparsely gray pilose or subglabrous	[24]
Leaves	The width of leaves under the stem is up to 30 cm The terminal leaflet is 7–10 cm long and 4–7 cm wide, often lobed	The width of leaves under the stem is up to 20 cm The terminal leaflet are 6–12 cm long and 4–9 cm wide, often 3 lobed	The width of leaves under the stem is up to 22 cm The terminal leaflet are 5–10 cm long and 3.5–9 cm wide, 3 deeply lobed	[18, 24]
Fruits	Follicles are oblong, 8–14 mm long and 2.5–5 mm wide, hairy, a 2–3 mm long stalk at the base, and a short beak at the top	The follicles are 5–6 mm long and 3–4 mm wide and the lower part has a thin handle about 1 mm long	Follicles are on the carpel stalk, 7–8 mm long, 4 mm wide, and the top is nearly truncate covered with white pubescence	[18, 24]
Seeds	Seeds are oval and brown, with 2.5–3 mm long	Usually 2 seeds, about 3 mm long	Seeds are 3–4, about 3 mm long, oval and brown	[18, 24]

*Cimicifuga* has the characteristics of cold resistance and grows well in warm and humid climate. It is suitable for growth in slightly acidic or neutral humus soil [19]. Owing to its medicinal values, this herb is extensively cultivated in Asian and European countries (Table S1) [20].

### Taxonomy

*Cimicifuga* is rich in species diversity and there are 28 species in temperate zone of the northern hemisphere. Except the three varieties specified in ChP, 12 kinds of non-genuine *Cimicifuga* were involved in China, such as *Cimicifuga acerin* (Sieb.et Zucc.) Tanaka inBull (distributed in Sichuan, Hubei of China), *Cimicifuga simplex* Wormsk. (distributed in Sichuan, Gansu, Hubei of China), *Cimicifuga yunnanensis* Hsiao. (distributed in Yunnan, China), *Cimicifuga foetida* L. Var. *foliolosa* Hsiao. (distributed in Sichuan, Xizang of China), *Cimicifuga nanchuanensis* Hsiao. (distributed in Chongqing, China), *Cimicifuga acerina* (Sieb. et Zucc.) Tanaka f. *hispidula* Hsiao. (distributed in Henan, Shanxi, Hubei of China), *Cimicifuga brachycarpa* Hsiao. (distributed in Yunnan of China), etc. [19, 21]. Although widely used in some regions, research on their phytochemical and bioactive properties is still necessary to explore its clinical safety and efficacy, and consider whether it can be used as an official substitute.

## Traditional uses

### The medicinal efficacy of Cimicifugae Rhizoma

Cimicifugae Rhizoma has been used to treat different diseases caused by insect sting, bacteria or viruses, such as mumps, chickenpox, malaria, measles, acute and chronic pharyngitis since the Eastern Han Dynasty [4, 22]. As recorded in *Shennong's Classic of Materia Medica*, Cimicifugae Rhizoma could treat acute infectious diseases caused by bacteria or viruses (dispel warm diseases in TCM theories). Cimicifugae Rhizoma had an effect on tonsillitis and mumps in *Diannan Bencao* [23]. It was mentioned in Compendium of Materia Medica that Cimicifugae Rhizoma could treat a variety of acute and chronic throat inflammation (throat obstruction in TCM theories) characterized by redness, swelling and pain. Headache, abdominal pain, sore throat and toothache were effectively relieved by Cimicifugae Rhizoma in *Mingyi Bielu* [4]. It recorded in *Bencao Biandu* that leucorrhea abnormalities caused by infection or inflammation could be eliminated by Cimicifugae Rhizoma [24].

As a folk medicine, the tender stems of *Cimicifuga* are boiled in hot water and dipped in sauce for consumption; the tender leaves could be used as cold dishes or stir-fried dishes, which are mainly used to clear stomach heat, alleviate symptoms such as toothache, mouth and tongue sores, swelling and pain in throat [6]. Furthermore, the description of Yang-Invigorating efficacy was increased after the Tang Dynasty. Impotence and feet cold had been significantly alleviated after the treatment of Cimicifugae Rhizoma recorded in *Compendium of Materia Medica*. It had therapeutic effect on postpartum lochia recorded in *Qianjin Fang*. Cimicifugae Rhizoma was widely applied in haemorrhage, diarrhea and rectal prolapse resulting from long-term diarrhea recorded in *Yaolong Xiaopin* [4]. Cimicifugae Rhizoma was used to treat children's epilepsy (infantile wind epilepsy in TCM theories) in *Yaoxing Lun* and also had the effects of sedation documented in *Rihuazi Bencao* [24].

### Prescriptions associated Cimicifugae Rhizoma

In TCM theory, Cimicifugae Rhizoma was combined with other TCMs to give full play to their efficacy. For example, Cimicifugae Rhizoma has a good effect on treating vertigo with compatibility of *Polygonatum sibiricum* Red [17]. It also had been used in combination with *Atractylodes lancea* (Thunb.) DC. to alleviate abdominal distention [25]. Shengma Biejia Decoction could treat yang poisoning (similar to systemic lupus erythematosus) with flushed complexion and macula of the whole body in the *Synopsis of the Golden Chamber* in the late Eastern Han Dynasty [26]. In the Yuan Dynasty, Laoya San was applied to treat gingiva with erosion, pain, red and swelling (ulcerative gingivitis in TCM theories) documented in *Lanshi Micang* written by Li Gao. With the development of TCM system in the Song Dynasty, more prescriptions related to Cimicifugae Rhizoma appeared. The Shengma decoction in *Benshi Prescription* applied to the treatment of chest and breast pain, while in *Shengji Zonglu* cured abdominal fullness. *Yixue Guangbiji* and *Mobao Zhaiji Yanfang* both mentioned that the prescriptions containing Cimicifugae Rhizoma had the advantage on dysentery and metrorrhagia during the Ming Dynasty [27]. In the Qing Dynasty, Shengma Decoction had good activity on the treatment of severe headache accompanied with ringing in the head (thunder headache in TCM theories) recorded in *Yifang Jijie* [24].

In Qingwei Powder, Cimicifugae Rhizoma not only played a role in clearing heat and detoxifying to relieve symptoms such as stomach fire and toothache [28], but also used as a channel ushering drug to assist guiding various medicinal herbs to reach the affected site. The channel ushering ability of Cimicifugae Rhizoma was also described in Yunnan Southern Materia Medica [23]. The Buzhong Yiqi decoction was applied to treat short breath, weakness, chronic diarrhea, anal prolapse and uterine prolapse caused by deficiency of spleen qi and stomach qi recorded in *Piwei Lun* [29]. Cimicifugae Rhizoma, as an assistant drug, exerted Yang-Invigorating efficacy to assist sovereign drug lifting the sinking middle qi. Moreover, 41 prescriptions related to Cimicifugae Rhizoma in different dynasties were summarized (Table S2). Since ancient times, the prescriptions of Cimicifugae Rhizoma have played an irreplaceable role in TCM. Therefore, we need to further comprehensively explore the efficacy and mechanism between the various medicinal materials in order to make it more safer and clearer for clinical application.

### Processing

Different processing methods are applied to enhance the efficacy, change the drug properties and meet clinical needs. Many processing methods of Cimicifugae Rhizoma have been developed (Table 2) and widely used in ancient and modern clinical practice. During the Eastern Tsin Dynasty, stir-frying with honey of Cimicifugae Rhizoma was described in the *Zhouhou Beiji Fang*. The honey products increased the efficacy of arresting sweating, cough and invigorating spleen-stomach and replenishing qi [30]. In the Northern and Southern Dynasties, processing with Polygonati Rhizoma juice, cleansing and steaming of Cimicifugae Rhizoma were recorded in *Leigong Paozhi Lun*. The purpose of cleansing was to remove impurities containing non-medicinal parts and sediment [31]. In the Song Dynasty, Cimicifugae Rhizoma's cutting methods were further refined, including mashing, fine grinding, filing, etc. Cutting was conducive to effective ingredients for decocting, subsequent processing and clinical dose adjusting. In addition, charcoal stir-frying and roasted Cimicifugae Rhizoma were documented in the same period [32]. Until the Ming and Qing Dynasties, the related processing methods of Cimicifugae Rhizoma were more enriched, including stir-frying with honey, stir-baked to yellow, vinegar-processed, wine-processed, salt-processed and ginger juice processed. Honey stir-frying and charcoal stir-frying of Cimicifugae Rhizoma are commonly applied in modern clinicals [29]. Raw products are better at promoting skin eruption, relieving the exterior, clearing heat and detoxifying; while honey-processed Cimicifugae Rhizoma are more effective in replenishing qi and elevating yang.

**Table 2:** Processing methods of Cimicifugae Rhizoma in different periods

Dynasty	Processing method	Book	Editor	References
Eastern Tsin dynasty	honey processed	Zhouhou Beiji Fang	Ge Hong	[31]
Northern and southern dynasties	Steam processed, cleansing, polygonati rhizoma juice processed	Leigong Paozhi Lun	Lei Xiao	[31]
Song dynasty	Cutting (mashing, fine grinding, filing) carbonized processed, roasted processed	Shengji Zonglu	Compiled by the government of the Song Dynasty	[30]
Ming dynasty	fried yellow processed, vinegar processed, wine processed, salt-water processed,	Pujifang Paozhi Dafa	Zhu Su Xi Yong	[24, 31]
Qing dynasty	Ginger juice processed, soil processed	Leizheng Zhicai Yizong Jinjian	Lin Peiqin Wu Qian	[24, 31]
Modern	Honey processed, wine processed, carbonized processed, etc	**/**	**/**	[24, 31]

TCMs processing is beneficial to increase the therapeutic effect, change drug properties and reduce toxic side effects to meet the needs of clinical medication. Processing is artificially altering the chemical composition based on their intended use and is a recreation of the quality of medicinal materials. Therefore, the quality evaluation is also necessary for the processing products. The content of caffeic acid, ferulic acid (FA), isoferulic acid (IFA) in stir-frying and wine-processed *C. dahurica* was significantly different from that in raw by ultra-performance liquid chromatography-tandem mass spectrometry (UHPLC-MS/MS). Although the IFA content decreased after processing, FA increased. In addition, the three phenolic acids were more easily absorbed after processing [33]. This conclusion was also confirmed by headspace solid phase microextraction together with gas chromatography-mass spectrometry (GC-MS) in *C. foetida* [34]. Through formalin-induced pain response, hot-plate test, acetic acid writhing test, the analgesic and sedative activities of honey-processed *C. dahurica* and *C. foetida* were significantly stronger than those of raw production [35]. The difference of pharmacological activity before and after processing of Cimicifugae Rhizoma might be attributed to the content of components. All of the above proved the differences in composition, content and efficacy before and after processing of Cimicifugae Rhizoma. In order to better apply to the clinic, the study on the differences between raw and processed products should be further deepened.

## Phytochemistry

**Fig. 1:**
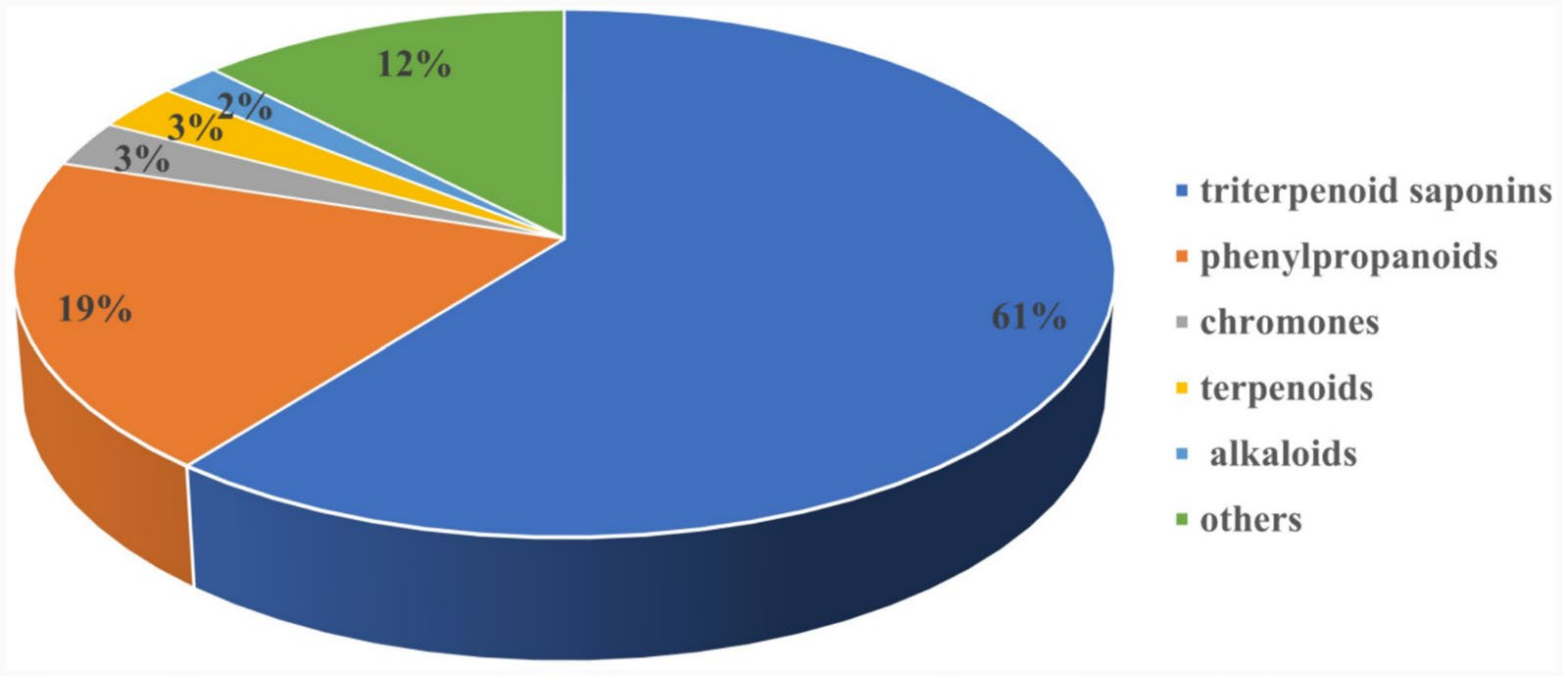
Percentages of different classes of pure compounds distributed in Cimicifugae Rhizoma

Recently, approximately 348 components have been isolated from Cimicifugae Rhizoma, including 211 triterpenoid saponins (1–211), 67 phenylpropanoids (212–278), 10 chromones (279–288), 7 alkaloids (289–295), 10 terpenoids (296–305) and 43 others (306–348). The proportion of different types of compounds in Cimicifugae Rhizoma as shown in Fig. 1. Among these, triterpenoid saponins are considered to be characteristics constituents and primary biologically-active phytochemicals. The related information about these components is summarized (Table S3) and structures of the relevant compounds are drawn by Chem Draw.

### Triterpenoid saponins

Cycloartane triterpenoids are the characteristic ingredients of Cimicifugae Rhizoma, which play an important role in anti-tumor, anti-viral, neuroprotective, hypolipidemic, etc. [36]. Due to the strong biological and pharmacological activities, it has become a research hotspot in recent years. The tetracyclic triterpenoids are formed by the chair-boat–chair conformational cyclization of epoxy squalene. The methyl group at C-10 is dehydrogenated with C-9 to form a ternary ring. The side chain at the C-17 is mostly an open chain with 8 carbons and its hydroxyl groups are dehydrated to form ketals or semi ketals. The structures mostly are dehydrated with xylose, glucose or arabinose to form monoglycoside, while disaccharides or trisaccharide glycosides are also present [21]. There may be hydroxyacetylated sugars on the structure that are mostly connected at the C-3 position.

According to the structural differences, these chemical constituents can be divided into several types, including cimigenol-type, hydroxyshengmanol-type, 16,23-diketotype, shengmanol-type, cimiacerogenin-type, acteol-type, foetidonol-type, cimilactone-type etc. [16, 21]. The majority is cimigenol-type and its structural characteristics is a double oxygen ring structure at 16:23–16:24. There is often a hydroxyl group substituted at the C-15 position. The feature of hydroxyshengmanol-type is that hemiketal structure at 16:24 is hydrolyzed and C-16 is substituted by a hydroxyl group. There are only 27 carbons in the parent nucleus of foetidonol-type and C-16 is connected with C-24 to form a six-membered carbon ring [1, 21]. The parent nucleus of cimilactone-type is one less of CH_2_ compared with foetidonol-type. In addition to the above categories, there are D-ring opening [37], side chain breaking [38] and a small amount of pentacyclic triterpenoids [39] (Fig. S1).

### Phenylpropanoids

The phenylpropanoids in Cimicifugae Rhizoma mainly include phenylpropionic acid and its derivatives (Fig. S2). Among them, FA (215), IFA (214) and caffeic acid (213) have been proven to have good antioxidant [40], antiviral [41], neuroprotective [42] and other pharmacological activities. Cimicifugaside A-E were isolated by means of HP-20 resin column, silica gel column and ODS CC column after 70% ethanol/H_2_O (*v*/*v*) extraction. Cimicifugaside A-B are two phenylpropionic acid compounds connected with D-allose while cimicifugaside C-E are derivatives formed by dehydration and condensation of FA with guaiacylglycerol [43]. Compounds 261–270 are phenolic amide glycosides characterized by two C6-C3 FAs linked by amide groups [44].

Lignan compounds in Cimicifugae Rhizoma are divided into four categories according to their characteristics and structural differences. The components of 271–273 are furofurans with methoxy and D-allose substitutions on the benzene ring. (−)-syringaresinol (274), (+)-isolarisiresinol 3-*O*-*β*-D-glucoside (275), syringaresinol di-*O*-*β*-D-allopyranoside (276) belong to dibenzylbutane, arylnaphthalene, dibenzyltyrolactone, respectively [45].

### Chromones

The primary type of chromones is furan chromones (Fig. S3). The methoxy group, hydroxyl group and glucose are the main substituents of these compounds. Cimifugin (283), norcimifugin (284) and prim-*O*-glucosylcimifugin (285) possessed high contents in C. *foetida* with strong anti-tumor, anti-inflammatory and antiviral properties [46–48]. Cimifugin-4'-*O*-[6''-feruloyl]-*β*-D-glucopyranoside (288) was a novel compound isolated from the acetone extract of *C. foetida*. Its structure was formed by a single glucose connecting two parts of cimifugin and FA [49].

### Alkalodis

Nowadays, there are seven alkaloids discovered from Cimicifugae Rhizoma (Fig. S4). Two indole alkaloids (289 and 290) were separated from *C. dahurica* and inhibited soluble epoxide hydrolase in a dose-dependent manner [50]. Two dimeric prenylindole alkaloids, named cimicifoetone A and cimicifoetone B, could not only be used as black dyes but also remarkably repress cancer cells multiplication [51].

### Terpenoids

Four monoterpene lactones (296–299) were obtained in *C. foetida*. Four nor-sesquiterpenoid glycosides (300–303) were identified from the n-butanol layer of *C. dahurica* [52]. A pinene monoterpene compound paeoniflorin (304) and an iridoid compound geniposide (305) were isolated from the 70% ethanol/H_2_O (*v*/*v*) of *C. dahurica* [44].

### Others

In addition to the above compounds, phenolic glycosides [53], aromatic carboxylic acids, quinone compound, flavonoids [52] and oligosaccharides [54] were reported from Cimicifugae Rhizoma. Furthermore, macromolecular triterpenoid-chromone hybrids (341–348) were identified from *C. foetida* by Nuclear Magnetic Resonance (NMR) and high resolution electrospray ionization mass spectroscopy (HRESI-MS) shown in Fig. S5 [55]. The macromolecular triterpenoid-chromone hybrids were isolated and identified from Cimicifugae Rhizoma, providing a material basis for the follow-up research for the activity of the herb and clinical application.

## Pharmacological activities

**Fig. 2:**
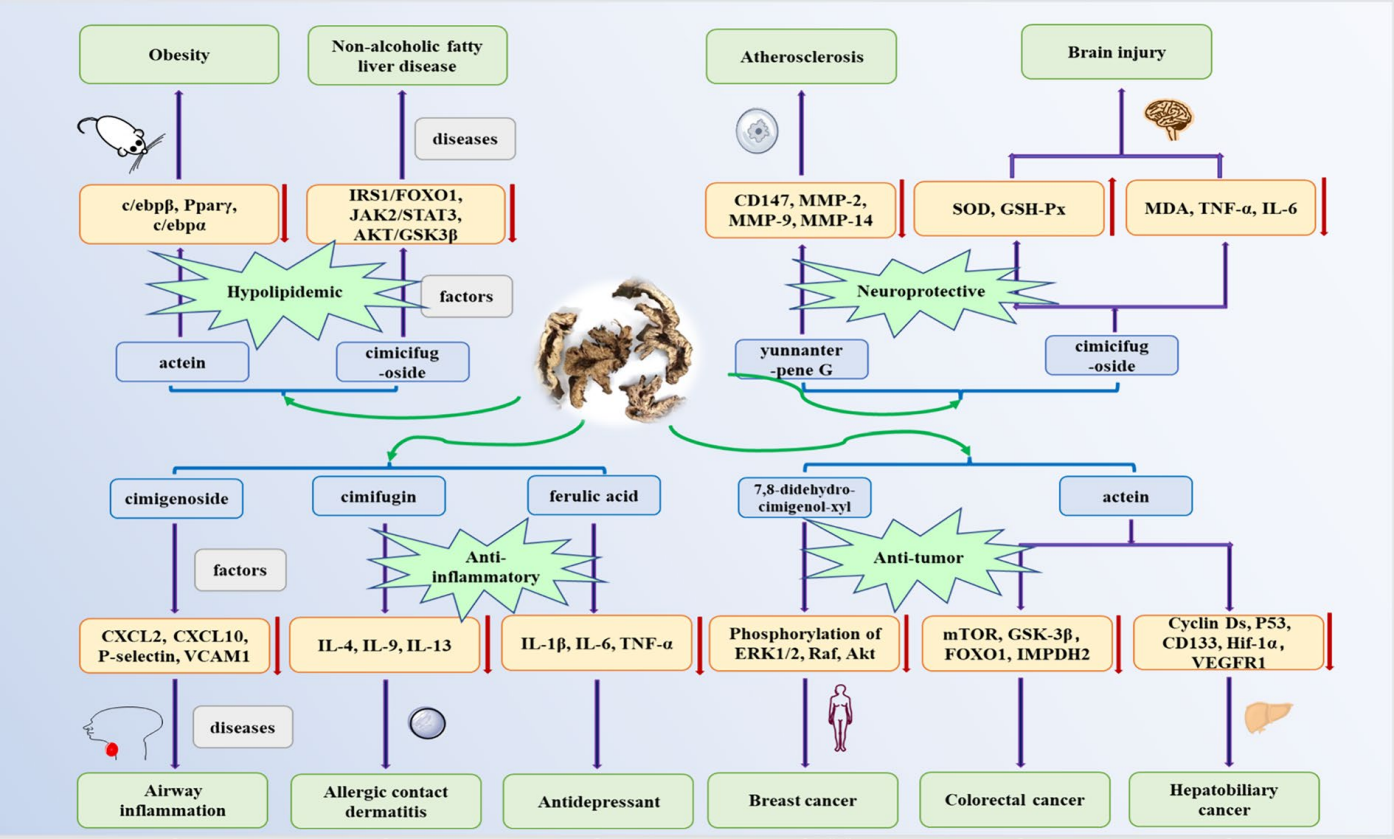
Changes in biological indicators of Cimicifugae Rhizoma on anti-inflammatory, anti-tumor, hypolipidemic and neuroprotective activities

Modern research showed that Cimicifugae Rhizoma had anti-inflammatory, antipyretic, analgesic, anti-ulcer effects, etc. [56]. Novel pharmacological activities on vasodilator [57], antioxidant, antiosteoporosis [58] and hypolipidemic had also been discovered in recent years. Triterpenoid saponins from Cimicifugae Rhizoma were considered to be the main active ingredients with multiple pharmacological effects. It was usually applied to relieve the symptoms of endocrine disorders, hypertension, osteoporosis, hyperlipemia, and depression [59–61]. The pharmacological activities of Cimicifugae Rhizoma were summarized according to the extraction, dosage and experimental model (Table S4). Related changes in biological indicators of Cimicifugae Rhizoma were shown in Fig. 2. The follow is an overview of pharmacological activities on the crude extracts and ingredients from Cimicifugae Rhizoma.

### Anti-inflammatory

Inflammation is considered as toxin of exogenous disease which is treated with heat-clearing and detoxifying herbs during the TCM. Cimicifugae Rhizoma has a strong anti-inflammatory effect due to its main efficacy in heat-clearing and detoxifying [62]. The 70% ethanol/H_2_O (*v*/*v*) of *Cimicifuga spp.* (30 mg/kg, 100 mg/kg) exerted the anti-inflammatory activity by inhibiting asthma response and repressing NF-kappa B (NF-*κ*B) phosphorylation, specific immunoglobulin E and Matrix Metalloproteinase 9 (MMP-9) expression in an allergic airway inflammation model [63]. Triterpenoid saponins of Cimicifugae Rhizoma were considered as the primary active components responsible for the anti-inflammatory activity. Triterpenoid saponins acted the effects by repressing the expression of Tumor Necrosis Factor alpha (TNF-*α*), Interleukin-12p40, Interleukin-6 (IL-6) in bone marrow-derived dendritic cells and inducible nitric oxide synthase, cyclooxygenase-2 (COX-2) in endotoxin-stimulated RAW264.7 cells [64]. As an active ingredient, cimigenol-3-*O*-*β*-D-xylopyranoside improved pulmonary inflammation through reducing poly-I:C stimulated neutrophil chemokine, cell adhesion molecules expression and inhibiting neutrophils infiltration into lung tissue [65]. Actein evidently relieved liver lipid accumulation, inflammation and fibrosis by increasing insulin and leptin resistance [36].

Phenolic acids and chromones of Cimicifugae Rhizoma also played roles in anti-inflammatory. Cimifugin (0.01, 0.1, 1 μg/mL) does-dependently reduced the production of proinflammatory mediators and inhibited allergic contact dermatitis [47]. The anti-inflammatory effect of cimiracemate A was performed by preventing extracellular regulated protein kinases phosphorylation and NF-*κ*B nuclear translocation [66]. FA inhibited the inflammatory response by regulating the expression of inflammatory markers heat shock protein 70, COX-2, TNF-*α*, IL-6, and Interleukin-1*β* (IL-1*β*) [67]. The underlying mechanisms was that FA inhibited the activation of microglia, NF-*κ*B signaling and NOD-like receptor thermal protein domain associated protein 3 in the prefrontal cortex [67].

### Anti-tumor

Breast cancer was considered to be the most usual invasive cancer in women [68]. Natural products with low toxicity and high efficiency had become the main source of drugs targeted to breast cancer. Studies had shown that Cimicifugae Rhizoma and its components remarkably repressed breast cancer cells proliferation. The *C. foetida* extract exerted anti-tumor effects by inhibiting the expression of heat shock protein-27 in MCF-7 breast cancer cells [69]. *C. dahurica* extract also embodied significant inhibitory effect on the MCF-7 cells proliferation based on the BrdU-proliferation test [70]. In addition, the ethyl acetate layer of *C. dahurica* inhibited the proliferation, migration and invasion of human breast cancer cells by up-regulating Bax, caspase-9/3 and cytochrome C and down-regulating Bcl-2 expression. The nude mice subcutaneous xenograft experiments further demonstrated the tumor growth inhibition [71]. The component analysis showed that triterpenoid saponins may be effective components against breast cancer, inducing cell apoptosis through the mitochondrial pathway [60, 71, 72]. The following were examples of the anticancer effect of monomer components. Cimigenoside (5 μM, 10 μM, 20 μM) induced apoptosis in breast cancer cells and inhibited presenilin 1 activity by down-regulating Notch intracellular domains expression in the nucleus, thereby repressing Notch protein cleavage and *γ*-secretase hydrolysis activity [72]. Actein showed strong anti-breast cancer effect in HCC1806 (IC_50_ = 2.78 μM) and MDA-MB-231 (IC_50_ = 9.11 μM) cell lines attributed to the acetylation of glycosyl hydroxyl at C-3, and the introduction of succinic acid structure at C-26 position of actein [73].

In addition, total glycosides of *C. dahurica* against hepatoma through inducing cycle arrest and apoptosis in HepG2 cells and inhibiting the implanted mouse H22 tumor growth dose-dependently [74]. It elevated the anti-hepatoma activity and also had the ability to inhibit the growth of human colon cancer cells when combined with cisplatin [75]. Actein and 26-deoxyactein performed significant anti-tumor activity in 12 human cancer cell lines with the IC_50_ range of 12.29 ~ 88.39 µg/mL in *vitro*. Moreover, the two compounds reduced the implanted mouse sarcoma S180 and human lung cancer A549 cells growth dose-dependently in *vivo* [76]. In particular, actein showed strong ability to induce apoptosis and suppress non-small-cell lung cancer growth when combined with iron oxide magnetic nanoparticles [77]. Cimigenoside (0, 1, 2 and 5 μmol/L) suppressed the activation of NF-κB signaling and inducing apoptosis in A549 cells [78]. Cimiside E and actein inhibited gastric cancer growth by activating p53/Caspase-3 signaling [79, 80]. Different doses (5, 15, 45 μM) of IFA were applied to treat blood cancer cells Raji, K562 and Jurkat by reducing cell viability, inhibiting cell growth and promoting apoptosis dose-dependently [81]. Actein, a compound with multiple activities, suppressed human colorectal cancer cell lines SW480 and HT-29 proliferation with IC_50_ values of 7.012 μM, 5.602 μM, respectively [82]. Other studies have shown that actein could promote oral squamous cell carcinoma cells apoptosis [83] and anti-hepatobiliary cancer [84]. The active ingredient of Cimicifugae Rhizoma possessed good anti-tumor effects and further studies in *vivo* and in *vitro* are necessary to explore its development into a potential anticancer drug.

### Antioxidant

As an effective antioxidant, Cimicifugae Rhizoma protected DNA and lipids from oxidative damage. The ethyl acetate layer of *Cimicifuga spp.* had a strong scavenging ability to DPPH and ATBS free radicals through metal chelation and free radical scavenging [providing hydrogen atoms (H•) and electrons (e)] [85]. The increase of phenolic content was positively correlated with antioxidant activity. The antioxidant activity of 2-feruloyl piscidic acid (IC_50_ = 9.33 μM) and 2-isoferuloyl piscidic acid (IC_50_ = 15.62 μM) was evaluated by DPPH free radical scavenging activity test and the content of phenolic acid was positively correlated with antioxidant activity [86]. Phenolic hydroxyl group of the benzene ring and a highly conjugated side chain in the opposite position benefited to the delocalization and stability of the phenoxy group in the whole molecule, which played a strong antioxidant capacity [87].

Caffeic acid, FA and IFA displayed positive correlations (the correlation coefficient values were 0.51, 0.50, and 0.51, respectively) with antioxidant levels by anti-lipidperoxidation, OH scavenging, Cu^2+^-chelating and Fe^3+^ reducing assays [88]. FA exerted antioxidant action by scavenging free radicals and resisting the damage of foreign substances. Studies have shown that FA reduced the oxidant content in the heart of arsenic-poisoned rats, restored antioxidant activity and also improved the effect of cadmium-induced oxidative damage in rat liver and kidney [40, 89]. The antioxidant activity of caffeic acid was quantitatively analyzed by intraperitoneal injection of 75 mg/kg Brdu in C57BL/6Ncr mice. The expression of 4-hydroxynonenal (an oxidative stress marker) was reduced after administration of caffeic acid (300 mg/kg) [42]. In summary, phenolic acids in Cimicifugae Rhizoma had antioxidant function due to its highly conjugated system. It is recommended to deeply explore the safety and reliability of phenolic acids in order to be developed into antioxidant drugs in the future.

### Antiviral

Cimicifugae Rhizoma had a good antiviral activity, which confirmed its anti-hepatitis B virus (HBV), human immunodeficiency virus (HIV) and other activities through pharmacological experiments. 60 patients with chronic hepatitis B were randomized into two groups to evaluate the anti-HBV activity of *C. foetida*. One group of patients was given adefovir (ADV) 10 or 30 mg every day. The other was treated with ADV and *C. foetida* (10 g were decocted and lyophilized, 100 mL/d, 3 times/d). It stimulated the inflammatory cytokines to inhibit HBV transcription and replication in patients [90]. *C. foetida* notably suppressed human respiratory syncytial virus (HRSV) in A549 (IC_50_ = 31.0 μg/mL) and HEp-2 (IC_50_ = 67.3 μg/mL) cell lines by plaque reduction test. The formation of HRSV plaque was effectively suppressed with time-dependent after *C. foetida* extract inoculation on HEp-2 cell line [91]. *Cimicifuga spp.* (100 μg/mL) was applied to DBT cells infected by coronavirus mouse hepatitis virus-A59 and Vero cells infested by porcine epidemic diarrhea virus or vesicular stomatitis virus. Titers of the above were 0.0044 ± 0.0029, 4.7 ± 1.2, 12.2 ± 3.6 respectively with strong antiviral activity [92]. Cimicifugae Rhizoma might play a specific inhibitory role by inhibiting RNA polymerase or other proteases that were essential for coronavirus RNA replication.

Total phenolic acid (20 mg/kg/d) markedly reduced the levels of hepatitis B surface antigen and hepatitis Be antigen in mice serum. The level of HBV DNA in cytoplasm core particles of HepG2-2.2.15 cell line also decreased [41]. Different doses (5, 50, 500 μM) of FA and IFA reduced Macrophage Inflammatory Protein 2 (MIP-2) level produced by infected RAW264.7 cells dose-dependently [93]. Afterwards, IFA (0.5 mg/d) promoted the survival rate in mice with lethal influenza virus pneumonia attributing to MIP-2 reduction and neutrophil aggregation. Due to the low mortality and weight changes in IFA group, it would be the potential drugs to treat pathogenic influenza virus pneumonia [94]. Screening anti-enterovirus A71 active drugs from 75 kinds of TCM and confirming that *C. heracleifolia* had strong activity (EC_50_ = 17 μg/mL, EC_90_ = 66 μg/mL, EC_50_: the concentration of compounds with 50% reduction in viral cytopathic effect). Fukinolic acid and cimicifugic acid A were obtained attributing to the activity-guided separation methods. According to the above structural characteristics, it was speculated that p-hydroxy group of benzene ring might play a therapeutic role [95]. Cimifugin displayed anti-HRSV effect by stimulating epithelial cells to secrete IFN-*β* fighting against viral infection while inhibiting virus adhesion and internalization [56]. Actein remarkably repressed HIV replication in H9 lymphocytes with EC_50_ of 0.375 μg/mL and the treatment index value of 144 [96]. In other words, Cimicifugae Rhizoma had a wide range of antiviral activities. The total phenolic acid of Cimicifugae Rhizoma was its active part for the treatment of viral hepatitis B due to specific phenolic hydroxyl structure. The structure–activity relationship of these compounds need to be strengthened so as to carry out more in-depth research.

### Relieving menopausal symptoms

Menopause, a period of transition from childbearing to old age and gradual decline of ovarian function [97]. Hormone/estrogen replacement therapy increased the risk of stroke and breast cancer during treatment if used for a long time [98]. Therefore, a safer and more effective method was urgently explored to reduce cancer incidence. Ximingting Tablets (XMT) mainly triterpene saponins of *Cimicifuga spp.* was developed to treat perimenopausal symptoms including depression. XMT (2.5 mg/time, 3 times/d) markedly improved menopausal symptoms but did not change hormone level and endometrial thickness [8]. Compared with estrogen therapy, XMT had a certain effect on menopausal symptoms with less side effects, which was worthy of promotion and application [99]. The similarity of *C. heracleifolia* and black cohosh was evaluated in relieving menopausal symptoms through constructing a KEGG pathway network. It was found that the administration group had a significant effect on lipid metabolism pathways, especially alpha-linolenic acid metabolism, fatty acid metabolism and biosynthesis of unsaturated fatty acids. In brief, *C. heracleifolia* was similar to black cohosh in relieving menopausal symptoms, and it could be considered as an ideal substitute for black cohosh in clinical application [100].

### Neuroprotective

The ethanol extract of *C. dahurica* could significantly inhibit A*β* production in APP-CHO cells, showing a protective effect on memory impairment [101]. 4'-methoxyl-3'-hydroxy-carboxybenzoyl isoferulic acid anhydride (10 μM) had strong neuroprotective activity compared to curcumin (a widely recognized strong antioxidant). The effect might be achieved through two conjugated ketone functional groups [87]. Cimicifugoside H-1 had a certain protective effect on neurons in ischemic brain tissue related with passing through the blood–brain barrier. It regulated the dysfunction of excitatory amino acid neurotransmitters in cerebral ischemia [102].

Cimiciphenone (IC_50_ = 16.7 μM) had a significant inhibitory effect on acetylcholinesterase (AchE). The formation of hydrogen bonds between cimiciphenone and the phenolic hydroxyl group of Tyr337, the active site of AchE, blocked the catalytic reaction. (*E*)-3-(3'-methyl-2'-butenylidene)-2-indolinone (IC_50_ = 13.8 μM) and (*E*)-3-(3'-methyl-2'-butenylidene)-1-methyl-2-indolinone (IC_50_ = 6.5 μM) applied to form hydrogen bonds with the binding site Asn 228 on butyrylcholinesterase, respectively. The above components were all potential cholinesterase inhibitors [103]. Prim-*O*-glucosylcimifugin (7.5 mg/kg, 15 mg/kg, 30 mg/kg) improved brain Cerebral ischemia/reperfusion (I/R) symptoms and neuronal apoptosis according to the number of errors in the step-down test and the neurological deficit score. It protected the brain from I/R injury in rats by reducing the levels of oxidative stress, inflammatory factors and activating the phosphatidylinositol 3-kinase/protein kinase B (PI3K/Akt) signal pathway [104]. In addition, caffeic acid (300 mg/kg) reduced activated microglia expression in the hippocampus of C57BL/6Ncr male mice to play a neuroprotective role compared with the vehicle group [42]. All of the above proved the Cimicifugae Rhizoma application in neuroprotection and provided ideas for subsequent research.

### Anti-angiogenesis

Cardiovascular disease, including thrombosis induced by platelet aggregation, is one of the main causes of death worldwide. Under certain conditions, such as pregnancy, the body may produce neovascularization and other conditions may lead to specific diseases, including cancer, rheumatoid arthritis, diabetic retinopathy [105]. Triterpenoid saponins in *C. foetida* had potential antithrombotic ability through screening in TCMSP database and docking with Y2Y_1_R protein in MRS2500 bag [106]. Different doses (0, 2, 4, 8 mg/mL) of Shengma Biejia Decoction (SMBJT) were applied to the chicken chorioallantoic membrane and tumor-bearing acute myeloid leukemia mouse models, respectively. SMBJT reduced microvessel density (MVD), immunohistochemical targeting CD31 and vascular endothelial growth factor receptor 2 expression to repress PI3K/Akt pathway targeted anti-angiogenesis [107].

Different doses of actein and 26-deoxyactein inhibited angiogenesis through depressing MVD and microvessel distribution on A549 (10, 30 mg/kg) and S180 (3, 9, 27 mg/kg) tumor cells or inducing G1 phase block in human leukemia HL-60 cells (6.25 ~ 25 µg/mL) [76]. FA (0 ~ 40 μM) had an effect on fibroblast growth factor receptor 1-induced phosphatidylinositol 3-kinase (PI3K)-protein kinase B (Akt) pathway, resulting in repressing the proliferation of melanoma cells and angiogenesis [108]. Cimicifugamide enhanced myocardial contractility and relieved heart failure symptoms through activated *β*-ARs in the toad heart failure model. It alleviated heart failure, induced hypothermia and promoted sweating in a mouse model for fever and sweating [109] The antiangiogenic effect of Cimicifugae Rhizoma had confirmed its efficacy in anti-tumor (inhibition of tumor angiogenesis) and relieving menopausal symptoms (repression of sweating and hypertension). It has been suggested that to carry out the adverse reaction of Cimicifugae Rhizoma in anti-angiogenesis, so as to safely apply it to the screening of other pharmacological activities on this base.

### Hypolipidemic

Abnormal lipid levels in the blood might cause fatty liver, diabetes, cardiovascular and other related diseases, which greatly threatened human health [110]. In recent years, some components of Cimicifugae Rhizoma could significantly improve dyslipidemia in mice and play a protective role. Body weight, epididymal and subcutaneous fat mass were decreased after administrating different doses of actein (10, 20, 30 mg/kg) in a high-fat diet-mice. Moreover, actein relieved the symptoms of hyperinsulinemia, hyperleptinemia and dyslipidemia had a good protective effect on nonalcoholic fatty liver disease in mice [36]. The inhibition rate of 23-*epi*-26-deoxyactein, cimicifugoside H-3, cimicifugoside H-2 in 3T3-L1 cells were 8.35% ~ 12.07% at 10 μM, repressing cells differentiation to achieve lipid-lowering effect [111]. Different doses (0.1 ~ 50 μM) of 23-*epi*-26-deoxyactein inhibited differentiation and lipid accumulation of 3T3-L1 cells dose-dependently by down-regulating the expression of C/ebp*β*, Ppar*γ*, C/ebp*α*. Besides, the addition of 23-*epi*-26-deoxyactein repressed fat deposition and adipocyte volume in high-fat diet-mice [112].

### Anti-osteoporosis

Osteoporosis is a common bone metabolic disease, systemic bone diseases caused by bone mineral density and bone mass decrease, bone microstructure destruction and bone fragility increase [113]. The incidence rate of women was higher due to the estrogen level dropped after menopause affecting cytokines and growth factors production in bone marrow [114]. The mixed extract of edible *Angelica sinensis* and *Cimicifuga spp.* (OAH19T) was often used for treating arthritis in the Far East: OAH19T (10, 20, 40 μg/mL) protected chondrocytes through down-regulating the levels of MMP-1, MMP-3, aggrecanase-1, aggrecanase-2 and promoting cell proliferation [115]. *C. heracleifolia* extract (0.1, 1, 10 μg/mL) enhanced the proliferation and osteogenic differentiation of human mesenchymal stem cells isolated from periodontal tissue [58]. Actein (0.1, 1 μM) induced the differentiation of MC3T3-E1 cells by promoting collagen content, alkaline phosphatase (ALP) activity, osteocalcin release and calcium deposition. Furthermore, actein inhibited the production of TNF-*α* and oxidative stress and protected osteoblasts by improving mitochondrial function in the presence of antimycin A [116] and 26-deoxyactein (0.1 ~ 1 mM) had the same effect [117]. Therefore, both might be good candidate drugs to protect osteoblast damage and dysfunction induced by oxidative stress.

#### Other pharmacological activities

XMT (20, 40, 80 mg/kg/d) could reduce the levels of plasma adrenocorticotropic hormone, serum corticosterone and adrenal weight in rats with chronic stress test with obvious antidepressant effect [118]. FA (20, 40, 80 mg/kg) increased the preference of mice for sucrose and shortened the immobility time of mice induced by unpredictable mild stress. It might reduce the expression of pro-inflammatory cytokines by inhibiting microglia activation to play an antidepressant role [67]. 96 early postmenopausal women were collected in clinical to study the differences between *C. foetida* (33.3 mg/d) and different hormones in relieving breast pain during this period. The total pain rate of the *C. foetida* group was vastly lower than hormone groups, demonstrating analgesic effect in *C. foetida* [119]. Cimilactone A had good anti-complementary activity with an IC_50_ value of 28.6 μM. The structure of its pharmacological action might be the C-23 terminal ketone [120]. Twenty-five compounds with significant antimalarial activity were screened out from *Cimicifuga spp.* and their EC_50_ values were 1.0 ~ 3.0 μM. According to the common structural characteristics, the 16,23:23,26:24,25-triepoxy played a therapeutic role [22].

The morphology of human gingival stem cells was slightly round and the number decreased when co-cultured with *C. heracleifolia* at high concentration (≥ 100 μg/mL). It was necessary to pay attention to the control of concentration and use time of *C. heracleifolia* to obtain better curative effect [121]. Cimigenol-3-*O*-*β*-D-xyloside decreased *β*-hexosaminidase release in HMC-1 cells due to the effect of xylose on the skeleton in inducing mast cell degranulation [61]. It might act on the AMP-activated protein kinase to achieve weight loss in mice [122]. Further research on the efficacy and mechanism of Cimicifugae Rhizoma extract in hypoglycemic effect need to be carried out. *C. dahurica* showed strong antibacterial activity by acting on E Coli, S. aureus, S. epidermidis and K. pneumoniae (MIC = 12.50 ~ 25.00 mg/mL). Furthermore, it effectively removed foreign objects from the wound and improved the healing ability (healing rate = 97.75 ± 0.76%) in skin injury mice [9]. *Cimicifuga spp.* extract had a significant inhibitory effect on cytotoxicity induced by ultraviolet irradiation [123]. FA (0 ~ 50 μM) co-cultured with human dermal fibroblasts irradiated by ultraviolet irradiation. It promoted cell proliferation, cycle progression and repressed the expression of MMP1 and MMP3 genes to protect the dermis [124]. *C. heracleifolia* (0 ~ 500 μg/mL) decreased the activation of MITF-tyrosinase and ERK-Akt signaling to inhibit melanin production. It had potential whitening value [125]. It illustrated the developmental value of Cimicifugae Rhizoma in the cosmetics industry and provided ideas for the development of Cimicifugae Rhizoma cosmetics.

The above effects, mechanism of Cimicifugae Rhizoma and its components in different pharmacological activities, suggested that they have good biological/pharmacological activity. Therefore, new components and pharmacological activities still need to be further explored.

## Quality control

The quality control of TCMs is the foundation for ensuring the effectiveness and safety of clinical medication. The identification methods of Cimicifugae Rhizoma including thin layer chromatography, microscopic identification, moisture, ash and impurity detection were described in ChP [3]. Meanwhile, it is stipulated that the content of IFA in the dry product shall not be less than 0.10%. However, Cimicifugae Rhizoma is a multi-original medicinal material with wide distributions and differences of the compositions as well as its contents. It is difficult to reflect the overall quality of Cimicifugae Rhizoma by single component. In recent years, the quality of Cimicifugae Rhizoma is evaluated from the perspectives of molecular biology, biological activity and multi-component quantification [126, 127]. The main analysis methods of Cimicifugae Rhizoma are DNA barcoding, HPLC-diode array detection, UPLC-photodiode array detector, UPLC-quadrupole time-of-flight mass spectrometry, GC-MS, UPLC with high-resolution accurate mass-mass spectrometry, UPLC-Q-TOF/HRMS^E^, etc. [34, 64, 86].

Accurate identification of TCMs species is the primary link in TCMs quality control. Scholars have continuously developed DNA barcoding to identify the species of Cimicifugae Rhizoma or adulterants in molecular level, followed by quality evaluation [128]. Based on the ITS regions, the melting temperatures, generated by nucleotide sequences, GC/AT ratio and length of the amplified product, significantly differentiated *C. foetida* (79.54 ℃), *C. heracleifolia* (82.48 ℃), *C. dahurica* (85.05 ℃), *C. acerina* (91.89 ℃) and *C. simplex* (91.43 ℃). Moreover, the presence of adulteration could be determined according to the changes in melting curve analysis[129]. ITS2, as a non-coding nuclear DNA, has the characteristics of easy sequence amplification, high success rate and strong universality. After amplification of the ITS2 nuclear gene sequence, a Neighbor-Joining tree was constructed and each species of Cimicifugae Rhizoma was divided into one branch, with significant inter species differences that can be distinguished from adulterants [130]. The ITS2 has been successfully used for the identification of Cimicifugae Rhizoma varieties [130–132].

It was reported that chemical differences analysis of original species is an important link in achieving comprehensive quality control. The HPLC fingerprints of *C. dahurica* and *C. heracleifolia* were established and IFA, cimifugin were characterized as the differential markers to differentiate the two species [131]. The metabolomics integrated chemometrics was used to screen the combinatorial discriminatory quality markers between *C. dahurica* and *C. foetida*. FA, IFA, cimifugin and caffeic acid were finally determined as the markers and the Fisher discriminant model was established according to the content of the four components for further differentiating the unknown original samples [127]. The Cimicifugae Rhizoma samples were classified into two groups by screening chemical markers including caffeic acid, FA, IFA, cimifugin, cimicifugic acid A and cimicifugic acid B based on UPLC-Q-Orbitrap MS/MS with multiple chemometric [126]. In generally, the compounds in phenylpropanoids and chromones deserved to be further screened as quality markers for Cimicifugae Rhizoma due to its remarkable specificity and content varieties between different species [133]. This may be laying the foundation for improving the quality evaluation system of Cimicifugae Rhizoma.

## Clinical studies

In modern times, Cimicifugae Rhizoma mainly worked in treating herpes zoster, oral ulcers, systemic lupus erythematosus, chronic pulmonary heart disease and other diseases [134]. So far, the efficacy varies with doses, and the therapeutic range has been expanded by skillful use of compatibility [135]. Xuanshen Shengma Decoction, combined with compound oxygen, reduced the inflammatory reaction of chronic cough in children and improved lung function [136]. Through clinical observation and analysis, children’s mycoplasma pneumonia was treated by Shengma Decoction combined with azithromycin. Moreover, it could reduce inflammatory response, improve clinical symptoms and immune function [137]. Shengma Gegen Decoction had good effect on treating herpes zoster [138]. Oral ulcer powder significantly improved the symptoms of ulcer in rats. Besides, it effectively treated and corrected the structural changes of ulcer mucosa caused by burning after combined with *Cimicifuga spp*. The two had synergistic effects so as to greatly shorten the healing time of ulcer and help to accelerate the healing of ulcer wounds [134]. Shengma Biejia Decoction had good therapeutic effect on systemic lupus erythematosus of yin deficiency and internal heat type [139].

*C. foetida* inhibited the abnormal gastrointestinal motility caused by senna leaves and exerted antidiarrheal effect due to the characteristics of Yang-Invigorating [140]. Shengma Gegen Decoction was used to treat chronic hepatitis B in immune clearance period through regulating the immune function of the body and inhibiting virus replication [141]. The treatment of chronic pulmonary heart disease with modified Mahuang Shengma Decoction could significantly improve the therapeutic effect. In addition, one patient with severe novel coronavirus pneumonia was cured after taking this prescription [142]. At present, Cimicifugae Rhizoma is mainly applied clinically in the form of prescription; therefore, it is necessary to speed up the mechanism of action, toxicity and side effects of Cimicifugae Rhizoma compatibility in order to play a safer and more effective role in clinical application.

## Toxicology

Cimicifugae Rhizoma was first recorded in *Sheng Nong’s Herbal Classic* and was listed as a superior product that was non-toxic. However, *C. dahurica* showed the toxic effects on the liver by co-culturing the rhizomes (IC_50_ = 1.417 mg/mL) and fibrous roots (IC_50_ = 1.26 mg/mL) of it with human hepatocytes L-02, respectively. Fibrous roots might cause more serious damage to the liver and female mice were more susceptible. In the 90 day sub-toxic study, compared with the rhizome group, the fibrous root group increased the levels of white blood cells, ALP, Alanine aminotransferase, total bilirubin, cholesterol, and the expression of *p*-NF-*κ*B dose-dependently. It was proven that long-term use of fibrous root could lead to serious liver damage, especially in women [143]. In a 13-week subchronic toxicity and genotoxicity study, *C. heracleifolia* extract (667, 2000 mg/kg) increased serum alanine transaminase activity and liver weights in female rats without effects on male [144]. Overall findings indicated that Cimicifugae Rhizoma extract may be prone to hepatotoxicity in female rats.

Moreover, the toxicity of the ingredients of Cimicifugae Rhizoma in vitro studies have been demonstrated on various tumor cells. Compounds including 25-*O*-acetylcimigenol, 20-*O*-acetylcimigenol-3-*O*-*β*-D-xylopyranosyl-3’-*O*-*β*-D-xylopyran-oside, 26-deoxy-acetylacteol-7(8)-en3-*O*-*β*-D-xylopyranosyl-3’-*O*-*β*-D-xylopyrano-side, cimilactone K showed promising cytotoxicities against the different cell lines mainly HL-60, SMMC-7721, A-549, MCF-7, SW480 with IC_50_ values ranging from 8.0 ~ 33.2 μM [49, 145]. Most of the reported adverse reactions of *Cimicifuga* were generally caused by black cohosh, such as dizziness, nausea, vomiting, headache, hepatitis, allergic reactions, acute liver injury, etc.[146, 147]. However, the incidence of adverse reactions was very low. Through clinical observation and analysis, XMT (3 tablets/d) could cause adverse reactions such as headache, palpitation, stomach discomfort. It might be related to estrogen receptor *β* which was affected by XMT in cardio-cerebrovascular and gastrointestinal [148]. Although a health product in people’s general perception, Cimicifugae Rhizoma in excess of the safe dosage may cause harm to the body. Moreover, there was no effective data on whether the current dosage of Cimicifugae Rhizoma was completely safe for clinical use. Further research is needed to elucidate its mechanism of action and safety.

## Conclusion and future perspectives

**Fig. 3:**
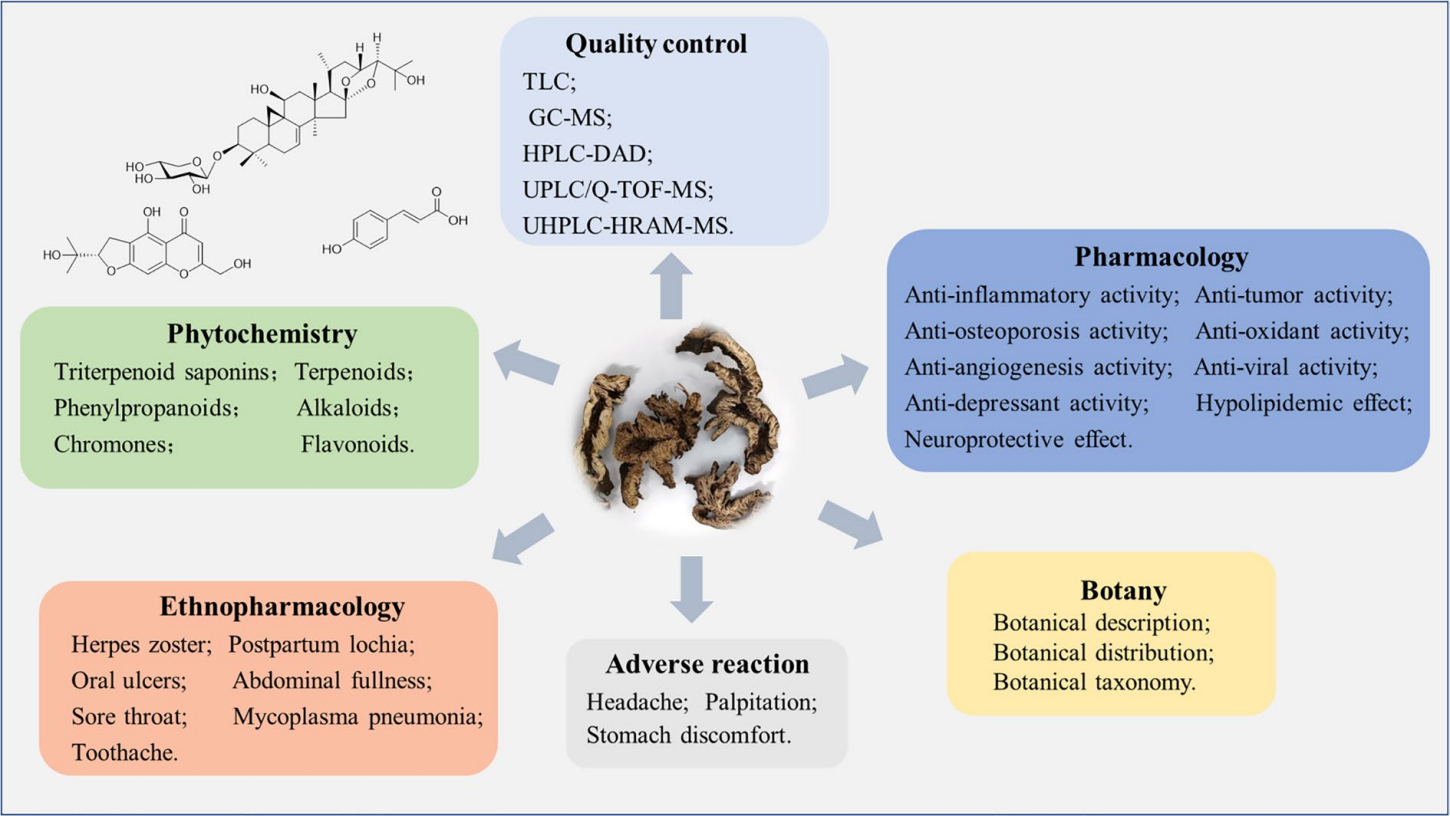
The schematic diagram of comprehensive review in Cimicifugae Rhizoma

The review aims at integrating a comprehensive information on Cimicifugae Rhizoma according to its botanic characterization, traditional uses, chemical components, pharmacological properties, quality control, toxicology and clinical studies (Fig. 3). Cimicifugae Rhizoma has been widely applied in clinical practice to treat headache, sore throat, abdominal pain, rectal prolapse, diarrhea and postpartum lochia for thousands of years. The chemical compositions of Cimicifugae Rhizoma serves as the basis for its clinical efficacy. Hitherto, 348 constituents have been isolated successfully from Cimicifugae Rhizoma, mainly triterpenoid saponins, phenylpropanoids, chromones, alkaloids, terpenoids and flavonoids. Cimicifugae Rhizoma extracts and its chemical constituents have been explored to extensive pharmacological activities including antitumor, anti-inflammatory, antioxidant, antiviral, analgesic and sedative effects etc. As for quality control, the ChP records that the content of IFA in Cimicifugae Rhizoma is not less than 0.10%.

Although the previous researches on Cimicifugae Rhizoma have achieved significant results, there are still many problems that we need to be resolved. Firstly, *Cimicifuga* species are rich in resources, but it also brings the phenomenon of mixed use and misuse among the same genus [149]. Accurately identify the species of Cimicifugae Rhizoma is an urgent problem to be solved. In order to make Cimicifugae Rhizoma play a better role in clinic, different original plants should be distinguished in terms of plant traits, types and contents of components, pharmacological activities as well as genes [126, 150]. In addition, only IFA is used as an indicator component in the content determination of Cimicifugae Rhizoma in ChP [3], while a single component is difficult to fully reflect the quality of Cimicifugae Rhizoma and does not conform to the overall concept of TCM. Therefore, it is essential to establish new detection methods or try to fill the gaps in quality control from the perspective of biological activity [151]. Secondly, the traditional uses of Cimicifugae Rhizoma mainly are applied in clinical practice to reflect therapeutic effects while they have not been proven by pharmacological researches. The recent studies about the pharmacological activity of Cimicifugae Rhizoma are limited to a single component like actein, cimigenoside, while TCM pays attention to the characteristics of ''multi-component, multi-target and overall regulation'' [152]. New pharmacological studies indicate that Cimicifugae Rhizoma has anti-enterovirus A71, anti-lung adenocarcinoma and lipid-lowering activities [57, 95, 153]. However, the mechanisms are not clear and clinical research has not been conducted. Accordingly, it is necessary to establish new methods to systematically evaluate the multiple active components of Cimicifugae Rhizoma, so as to study the synergistic effects of acting on multiple targets and achieve multiple target drug therapy. Thirdly, the exploration on the toxicity of Cimicifugae Rhizoma is insufficient and shallow due to the complexity of the components of Cimicifugae Rhizoma and the unpredictability of drug-body interactions. The safety evaluation of Cimicifugae Rhizoma is crucial because it is not only used in clinical practice, but also as a functional food in daily life [54]. Studies have shown that the adverse reactions are related to estrogen receptors *β* after taking XMT. Therefore, the safe dosage and toxic mechanism are needed to explore the adverse reactions of Cimicifugae Rhizoma or the safety of its medication.

Fourthly, the processing methods of Cimicifugae Rhizoma are numerous, including stir-frying with honey, stir-baked to yellow, vinegar-processed, wine-processed and so on [31]. However, the processing process is mostly based on personal experience and lacks standardized preparative techniques, making it difficult to ensure the consistency of the decoction pieces quality. Electronic nose, electronic tongue and electronic eye are used to achieve objective evaluation of the odor and appearance of the processed products in order to standardize the processing technology of Cimicifugae Rhizoma [154]. Meanwhile, quality evaluation indicators should be established by measuring the changes in the content of relevant components in the processed products. Moreover, the differential study of pharmacological activity and action mechanism between the raw and processed products was strengthened to better exert their clinical efficacy [155].

In general, it was summarized the morphological differences, traditional uses, chemical constituents, pharmacological activities, quality control and toxicology of Cimicifugae Rhizoma and prospected the future research directions for rational application in the future.

### Supplementary Information

Supplementry Material 1 Fig. S1 The structures of triterpenoid saponins in Cimicifugae Rhizoma (1-211). The structures of triterpenoid saponins in Cimicifugae Rhizoma (1-211). The structures of triterpenoid saponins in Cimicifugae Rhizoma (1-211). The structures of triterpenoid saponins in Cimicifugae Rhizoma (1-211). The structures of triterpenoid saponins in Cimicifugae Rhizoma (1-211). The structures of triterpenoid saponins in Cimicifugae Rhizoma (1-211). The structures of triterpenoid saponins in Cimicifugae Rhizoma (1-211).Fig. S2 The structures of phenylpropanoids in Cimicifugae Rhizoma (212-278). The structures of phenylpropanoids in Cimicifugae Rhizoma (212-278). The structures of phenylpropanoids in Cimicifugae Rhizoma (212-278). Fig. S3 The structures of phenylpropanoids in Cimicifugae Rhizoma (212-278). The structures of chromones in Cimicifugae Rhizoma (279-288). Fig. S4 The structures of alkalodis (289-295) and terpenoids (296-305) in Cimicifugae Rhizoma. Fig. S5 The structures of others in Cimicifugae Rhizoma (306-340). The structures of others in Cimicifugae Rhizoma (341-348). Table S1 Distribution of C. foetida, C. heracleifolia, C. dahurica. Table S2 The traditional uses of Cimicifugae Rhizoma in different periods are summarized. Table S3 Phytochemical constituents of Cimicifugae Rhizoma. Table S4. The pharmacological activities, extract, dose, model and results of Cimicifugae Rhizoma are summarized.

## Data Availability

The data supporting this review are from previously reported studies and datasets, which have been cited.

## Abbreviations

*C.** dahurica*: *Cimicifuga dahurica* (Turcz.) Maxim
*C.** foetida*: *Cimicifuga foetida* L.
*C.** heracleifolia*: *Cimicifuga heracleifolia* Kom
ChP: The 2020 edition of Chinese Pharmacopoeia
CNKI: The China National Knowledge Infrastructure
FA: Ferulic acid
GC-MS: Gas chromatography-mass spectrometry
HPLC-UV/MS: High performance liquid chromatography-ultraviolet/mass spectrometry
IFA: Isoferulic acid
I/R: Cerebral ischemia/reperfusion
OAH19T: Angelica sinensis and Cimicifugae Rhizoma
PI3K/Akt: Phosphatidylinositol 3-kinase/protein kinase B
SMBJT: Shengma Biejia Decoction
TCMs: Traditional Chinese Medicines
UHPLC-MS/MS: Ultra-performance liquid chromatography-tandem mass spectrometry
XMT: Ximingting tablets
