# Supplementary material for: Traditional uses, phytochemistry, pharmacology, quality control and clinical studies of Cimicifugae Rhizoma: a comprehensive review
Source: Chin Med. 2024 May 7;19:66. doi: 10.1186/s13020-024-00937-7 (PMC11075223; doi:10.1186/s13020-024-00937-7)
Supplement: Supplementary file 1 — Supplementry Material 1 Fig. S1 The structures of triterpenoid saponins in Cimicifugae Rhizoma (1-211). The structures of triterpenoid saponins in Cimicifugae Rhizoma (1-211). The structures of triterpenoid saponins in Cimicifugae Rhizoma (1-211). The structures of triterpenoid saponins in Cimicifugae Rhizoma (1-211). The structures of triterpenoid saponins in Cimicifugae Rhizoma (1-211). The structures of triterpenoid saponins in Cimicifugae Rhizoma (1-211). The structures of triterpenoid saponins in Cimicifugae Rhizoma (1-211).Fig. S2 The structures of phenylpropanoids in Cimicifugae Rhizoma (212-278). The structures of phenylpropanoids in Cimicifugae Rhizoma (212-278). The structures of phenylpropanoids in Cimicifugae Rhizoma (212-278). Fig. S3 The structures of phenylpropanoids in Cimicifugae Rhizoma (212-278). The structures of chromones in Cimicifugae Rhizoma (279-288). Fig. S4 The structures of alkalodis (289-295) and terpenoids (296-305) in Cimicifugae Rhizoma. Fig. S5 The structures of others in Cimicifugae Rhizoma (306-340). The structures of others in Cimicifugae Rhizoma (341-348). Table S1 Distribution of C. foetida, C. heracleifolia, C. dahurica. Table S2 The traditional uses of Cimicifugae Rhizoma in different periods are summarized. Table S3 Phytochemical constituents of Cimicifugae Rhizoma. Table S4. The pharmacological activities, extract, dose, model and results of Cimicifugae Rhizoma are summarized. [file 13020_2024_937_MOESM1_ESM.docx]

**Traditional uses, phytochemistry, pharmacology, quality control and clinical studies of Cimicifugae Rhizoma: A comprehensive review**

Qianqian Zhang^1,2^, Wei Wei^1,2,3^, Xingyue Jin^1,2^, Jin Lu^1,2^, Shujing Chen^1,2,3^, Omachi Daniel Ogaji^1,2^, Shaoxia Wang^1^, Kunze Du^1,2^, Yanxu Chang^1,2,3^, Jin Li^1*^

^1^State Key Laboratory of Component-based Chinese Medicine, Tianjin University of Traditional Chinese Medicine, Tianjin, 301617, China

^2^Tianjin Key Laboratory of Phytochemistry and Pharmaceutical Analysis, Tianjin University of Traditional Chinese Medicine, Tianjin, 301617, China

^3^Haihe Laboratory of Modern Chinese Medicine, Tianjin, 301617, China

*Corresponding author:

**Jin Li**, State Key Laboratory of Component-based Chinese Medicine, Tianjin University of Traditional Chinese Medicine, Tianjin, 301617, China

E-mail: Lijin@tjutcm.edu.cn (J. Li)

**Fig. S1 The structures of triterpenoid saponins in** **Cimicifugae** **Rhizoma (1-211)**

Fig. S1 The structures of triterpenoid saponins in Cimicifugae Rhizoma (1-211)

Fig. S1 The structures of triterpenoid saponins in Cimicifugae Rhizoma (1-211)

Fig. S1 The structures of triterpenoid saponins in Cimicifugae Rhizoma (1-211)

**Fig. S1 The structures of triterpenoid saponins in Cimicifugae Rhizoma (1-211)**

**Fig. S1** The structures of triterpenoid saponins in Cimicifugae Rhizoma (1-211)

**Fig. S1** The structures of triterpenoid saponins in Cimicifugae Rhizoma (1-211)

**Fig.** S1 The structures of triterpenoid saponins in Cimicifugae Rhizoma (1-211)

**Fig. S1** The structures of triterpenoid saponins in Cimicifugae Rhizoma (1-211)

**Fig.** **S2** The structures of phenylpropanoids in Cimicifugae Rhizoma (212-278)

Fig. S2 The structures of phenylpropanoids in Cimicifugae Rhizoma (212-278)

Fig. S2 The structures of phenylpropanoids in Cimicifugae Rhizoma (212-278)

Fig. S2 The structures of phenylpropanoids in Cimicifugae Rhizoma (212-278)

Fig. S3 The structures of chromones in Cimicifugae Rhizoma (279-288)

Fig. S4 The structures of alkalodis (289-295) and terpenoids (296-305) in Cimicifugae Rhizoma

Fig. S5 The structures of others in Cimicifugae Rhizoma (306-340)

Fig. S5 The structures of others in Cimicifugae Rhizoma (341-348)

Table S1 Distribution of C. foetida, C. heracleifolia, C. dahurica.

| Species | Distribution | Growing environment | Reference |
| --- | --- | --- | --- |
| *C. foetida* | Tibet, Yunnan, Sichuan, Qinghai, Gansu, Shaanxi, western Henan and Shanxi of China; Mongolia, Siberia of the Soviet Union. | Growing in the edge of mountain forests or roadside grass between 1700 and 2300 meters above sea level. | [1] |
| *C. heracleifolia* | Northeastern provinces of China, North Korea to the Far East of the Soviet Union. | Growing in the grass or bushes on the hillside. | [1] |
| *C. dahurica* | Shanxi, Hebei, Inner Mongolia, three northeastern provinces of China, the eastern Siberia, far east of the Soviet Union, Mongolia. | Growing in the shrub at the edge of the mountain forest and in the sparse forest or grassland on the hillside between 300-1200 meters above sea level. | [1] |

Table S2 The traditional uses of Cimicifugae Rhizoma in different periods are summarized.

| Dynasty | Classic | Writer | Preparation name | Traditional uses | Reference |
| --- | --- | --- | --- | --- | --- |
| Eastern Han Dynasty | *Treatise on Febrile Diseases* | Zhang Zhongjing | Mahuang Shengma Decoction | Treating cold hands and feet, increased frequency of urination, vomiting pus and blood. | [2] |
| Eastern Han Dynasty | *Synopsis of the Golden Chamber* | Zhang Zhongjing | Shengma Biejia Decoction | Treating blush, sore throat, salivary with pus and blood. | [3] |
| Tang Dynasty | *Qianjin Fang* | Sun Simiao | **/** | Treating oral fever and sores, heat arthralgia itching and postpartum lochia. | [3] |
| Tang Dynasty | *Qianjin Fang* | Sun Simiao | Huanglian Shengma Decoction | Treating sore in mouth. | [4] |
| Tang Dynasty | *Qianjin Fang* | Sun Simiao | Shengma cream | Treating erysipelas and heat sore. | [4] |
| Song Dynasty | *Shenghui Fang* | Wang Huaiyin | Shengma Pill | Treating throat occlusion. | [3] |
| Song Dynasty | *Shenghui Fang* | Wang Huaiyin | Shengma Powder | Treating physical pain, restless chest and lack of appetite. | [4] |
| Song Dynasty | *Yanshi Xiaoer Fanglun* | Yan Xiaozhong | Shengma Gegen Decoction | Treating typhoid fever, plague, headache, limb pain. | [3] |
| Song Dynasty | *Benshi Fang* | Xu Shuwei | Shengma Decoction | Treating lung carbuncle, vomiting pus blood, chest and breast pain, halitosis. | [3] |
| Song Dynasty | *Shengji Zonglu* | The dynasty | Lingyangjiao Shengma Decoction | Treating exogenous cold disease, anhidrotic convulsion. | [4] |
| Song Dynasty | *Shengji Zonglu* | The dynasty | Shengma Zhizhi Decoction | Treating excessive heat in stomach. | [4] |

continued

| Dynasty | Classic | Writer | Preparation name | Traditional uses | Reference |
| --- | --- | --- | --- | --- | --- |
| Song Dynasty | *Leizheng Huoren Shu* | Zhu Gong | Shengma Liuwu Decoction | Treating exogenous cold disease in July of pregnancy, high fever, reddish macules blackening, hematuria. | [4] |
| Song Dynasty | *Taiping Huimin Heji Jufang* | Chen Shiwen | Shengma Heqi Yin | Treating scabies originating from limbs, pain and itch of arm and hip, tinnitus and eye pain. | [4] |
| Song Dynasty | *Sanyin Fang* | Chen Yan | Shengma Chaihu Decoction | Treating deficiency and heat of heart and spleen, sore in tongue. | [4] |
| Song Dynasty | *Shanghan Zongbing Lun* | Pang Anshi | Huangbo shengma Decoction | Treating epidemic oral aphthae. | [4] |
| Yuan Dynasty | *Lanshi Micang* | Li Gao | Shengma Tuoli Decoction | Treating comedo nevus between breasts. | [4] |
| Yuan Dynasty | *Lanshi Micang* | Li Gao | Shengyang Jujing Decoction | Treating excessive menstruation, anemia, hematochezia, hematuria caused by functional uterine bleeding. | [4] |
| Yuan Dynasty | *Lanshi Micang* | Li Gao | Laoya San | Treating teeth chancre swelling pain, teeth shaken to fall, teeth yellow and halitosis. | [3] |
| Yuan Dynasty | *Piwei Lun* | Li Gao | Buzhong Yiqi Decoction | Treating qi deficiency subsidence, fever, speech weakness, spontaneous sweating. | [3] |
| Yuan Dynasty | *Waike Jingyi* | Qi Dezhi | Shengma Tazhong Decoction | Treating early growth of scabies. | [3] |
| Ming Dynasty | *Jingyue Quanshu* | Zhang Jiebin | Juyuan Jian | Treating qi deficiency subsidence, haemorrhage, yang qi serious dissipation [close](https://cn.bing.com/dict/search?q=Close&FORM=BDVSP6&cc=cn) [to](https://cn.bing.com/dict/search?q=to&FORM=BDVSP6&cc=cn) [death](https://cn.bing.com/dict/search?q=death&FORM=BDVSP6&cc=cn) . | [3] |

continued

| Dynasty | Classic | Writer | Preparation name | Traditional uses | Reference |
| --- | --- | --- | --- | --- | --- |
| Ming Dynasty | *Diannan Bencao* | Lan Mao | Shengma Decoction | Treating children's pox, fever headache, suppurative tonsillitis and mumps. | [3] |
| Ming Dynasty | *Douzhen Renduan Lu* | Xu Qian | Decoction for Removing Meridian Obstruction | Treating measles at the beginning, body heat without sweat, cough  and sore throat. | [3] |
| Ming Dynasty | *Medical Record* | Yu Tuan | Shengfa Erchen Decoction | Treating phlegm stagnation and dysuria. | [4] |
| Ming Dynasty | *Shenshi Yaohan* | Fu Renyu | Shengma Gange Decoction | Treating redness, swelling and pain in eyes,  aversion to cold with fever. | [4] |
| Ming Dynasty | *Zhunsheng Leifang* Volume V | Wang Kentang | Baizhu Shengma Decoction | Treating tetanus and limb spastic. | [3] |
| Ming Dynasty | *Yixue Rumen* Volume VI | Li Chan | Shengma Shunqi Decoction | Treating dietary inadequacy, shortness of breath and lacks luster of the face due to overanxious | [3] |
| Ming Dynasty | *Qixiao Liangfang* | Dong Su | Shengma Xieshi Decoction | Treating pharyngeal and diaphragmatic obstruction, abdominal pain, reversed qi and constipation. | [4] |
| Ming Dynasty | *Maiyin Zhenzhi* | Qin Changyu | Shengma Qingwei Powder | Treating teeth bleeding caused by excessive heat in stomach and gastrointestinal heat accumulation. | [4] |
| Ming Dynasty | *Renshu Bianlan* | Zhang Jie | Shengyang Yihuo Decoction | Treating taken too much cold medicine when eye disease leading to prolonged inability to cure. | [4] |
| Ming Dynasty | *Puji Fang* | Zhu Su | Huangqin Shengma Decoction | Treating pediatric headaches and physical pain caused by sudden changes in temperature. | [4] |

continued

| Dynasty | Classic | Writer | Preparation name | Traditional uses | Reference |
| --- | --- | --- | --- | --- | --- |
| Qing Dynasty | *Liangpeng Huiji* | Sun Wei | Fangfeng Shengma Decoction | Treating teethache. | [4] |
| Qing Dynasty | *Yifang Jijie* | Wang Ang | Shengma Decoction | Treating thunder headache, head and face swelling and pain. | [3] |
| Qing Dynasty | *Yixue Zhongzhong Canxi lu* | Zhang Xichun | Shengxian Decoction | Treating sinking of qi in the chest, shortness and insufficient of breath. | [3] |
| Qing Dynasty | *Yizong Jinjain* | Wu Qian | Shengma Xiaodu Yin | Treating impetigo. | [4] |
| Qing Dynasty | *Fuqing Zhunvke* | Fu Shan | Shengju Dabu Decoction | Treating postpartum haemorrhage. | [4] |
| Qing Dynasty | *Waike Dacheng* | Qi Kun | Shengju Decoction | Treating osteomyelitis of mandible; pharynx, larynx, intraaural swelling and pain. | [4] |
| Modern | *Chinese Pharmacopoeia 2020* | **/** | Qingshu Yiqi Wan | Treating heat stroke and sunstroke. | [5] |
| Modern | *Chinese Pharmacopoeia 2020* | **/** | Yiqi Congming Pill | Treating vision fainting, deafness and tinnitus | [5] |
| Modern | *Gan Zuwang Fang* | Gan Zuwang | Shengqing Liuqi Yin | Treating aero-otitis media. | [4] |
| Modern | *Zhao Zuozhong Fang* | Zhao Zuozhong | Shengge Erchong Decoction | Treating osteomyelitis. | [4] |

Table S3 Phytochemical constituents of Cimicifugae Rhizoma.

| Number | Chemical constituents | Molecular formula | MW | Source plants | Part of the plants | References |
| --- | --- | --- | --- | --- | --- | --- |
| **triterpenoid saponins** | | | | | | |
| 1 | 24-*epi*-7,8-didehydrocimigenol | C_30_H_46_O_5_ | 486.68 | *C. heracleifolia*/  *C. dahurica* | Rhizome | [6] |
| 2 | 25-*O*-acetyl-7,8-didehydrocimigenol | C_32_H_48_O_6_ | 528.35 | *C. heracleifolia*/  *C. dahurica* | Rhizome | [7, 8] |
| 3 | 7,8-didehydrocimigenol-3-*O*-*β*-D-xylopyranoside | C_35_H_54_O_9_ | 618.40 | *C. foetida*/  *C. dahurica*/  *C. heracleifolia* | Rhizome | [6, 9] |
| 4 | 7,8-didehydrocimigenol 3-*O*-*α*-L-arabinopyranoside | C_35_H_54_O_9_ | 618.40 | *C. dahurica* | Rhizome | [6] |
| 5 | 25-*O*-acetyl-7,8-didehrocimigenol 3-*O*-*β*-D-xylopyranoside | C_37_H_56_O_10_ | 660.40 | *C. dahurica* | Rhizome | [6] |
| 6 | cimiricaside B | C_36_H_56_O_9_ | 632.39 | *C. dahurica* | Root | [10] |
| 7 | (23*R*,24*R*)-16*β*,23;16*α*,24-diepoxy-12*β*-acetoxy-cycloart-7-en-3*β*,15*α*,25-triol 3-*O*-*β*-D-xylopyranoside | C_37_H_56_O_11_ | 676.40 | *C. foetida* | Rhizome | [11] |
| 8 | 12*β*-hydroxy-7,8-didehydrocimigenol-3-*O*-*β*-D-xylopyranoside | C_35_H_54_O_10_ | 634.37 | *C. foetida* | Rhizome | [12] |
| 9 | (23*R*,24*S*)-16*β*,23;16*α*,24-diepoxy-cycloart-7-en-3*β*,11*β*,25-triol 3-*O*-*β*-D-xylopyranoside | C_35_H_54_O_9_ | 618.38 | *C. foetida* | Rhizome | [11] |
| 10 | cimimanol D | C_40_H_62_O_13_ | 750.42 | *C. foetida* | Rhizome | [13] |

Continued

| Number | Chemical constituents | Molecular formula | MW | Source plants | Part of the plants | References |
| --- | --- | --- | --- | --- | --- | --- |
| 11 | 7,8-didehydro-25-dehydrocimigenol-3-*O*-*β*-D-xylopyranoside | C_35_H_52_O_8_ | 601.40 | *C. dahurica* | Rhizome | [6] |
| 12 | 1,7-diencimigenol-3,12-dione | C_30_H_40_O_6_ | 496.28 | *C. foetida* | Rhizome | [14] |
| 13 | 1-en-cimigenol-3,11-dione | C_30_H_42_O_6_ | 498.30 | *C. foetida* | Rhizome | [14] |
| 14 | 11*β*-hydroxy-7-en-cimigenol-3-one | C_30_H_44_O_6_ | 500.31 | *C. foetida*/  *C. heracleifolia* | Rhizome | [14, 15] |
| 15 | cimigenol-3-one | C_30_H_46_O_5_ | 486.33 | *C. foetida*/  *C. dahurica*/  *C. heracleifolia* | Rhizome | [8, 9, 16] |
| 16 | cimiricaside C | C_39_H_58_O_11_ | 702.31 | *C. dahurica* | Root | [10] |
| 17 | cimdahxynoside D | C_36_H_56_O_11_ | 664.38 | *C. dahurica* | Root | [17] |
| 18 | 7,8-didehydrocimigenol-3-*O*-*β*-D-galactopyranoside | C_36_H_56_O_10_ | 648.00 | *C. dahurica* | Root | [17] |
| 19 | cimdahxynoside E | C_36_H_56_O_11_ | 664.38 | *C. dahurica* | Root | [17] |
| 20 | 25-anhydro-7,8-didehydrocimigenol | C_30_H_44_O_4_ | 468.32 | *C. dahurica* | Root | [18] |
| 21 | 12*β*-hydroxycimigenol | C_30_H_48_O_6_ | 504.35 | *C. foetida* | Rhizome | [9, 16] |
| 22 | cimigenol | C_30_H_48_O_5_ | 488.35 | *C. foetida*/  *C. dahurica/*  *C. heracleifolia* | Rhizome | [8, 9, 19] |
| 23 | 25-*O*-acetylcimigenol | C_32_H_50_O_6_ | 530.36 | *C. foetida* | Rhizome | [9, 16] |

Continued

| Number | Chemical constituents | Molecular formula | MW | Source plants | Part of the plants | References |
| --- | --- | --- | --- | --- | --- | --- |
| 24 | 25-*O*-methyl-cimigenol | C_31_H_50_O_5_ | 502.37 | *C. dahurica/*  *C. foetida* | Rhizome | [15, 20] |
| 25 | 12*β*-acetoxycimigenol | C_32_H_50_O_7_ | 546.36 | *C. foetida* | Root | [21] |
| 26 | cimigenol-3-*O*-*α*-L-arabinoside | C_35_H_56_O_9_ | 620.39 | *C. dahurica/*  *C. foetida/*  *C. heracleifolia* | Root | [8, 21, 22] |
| 27 | cimigenoside | C_35_H_56_O_9_ | 620.80 | *C. dahurica*  *C. foetida*/  *C. heracleifolia* | Aerial part | [22, 23] |
| 28 | cimigenol-3-*O*-*β*-D-galactoside | C_36_H_58_O_11_ | 666.40 | *C. foetida* | Rhizome | [16] |
| 29 | 25-*O*-acetyl-12*β*-acetoxycimigenol-3-*O*-*β*-D-xylopyranoside | C_39_H_60_O_12_ | 720.41 | *C. Foetida*/  *C. dahurica* | Root | [8, 21] |
| 30 | 25-*O*-ethylcimigenol-3-*O*-*β*-D-xylopyranoside | C_37_H_60_O_9_ | 648.42 | *C. foetida* | Root/Rhizome | [24] |
| 31 | 12*β*-*O*-acetylcimigenol-3-*O*-*β*-D-  xyloside | C_37_H_58_O_11_ | 678.40 | *C. foetida* | Rhizome | [9, 16] |
| 32 | cimigenol-3-*O*-*β*-D-xyloside | C_35_H_56_O_10_ | 636.39 | *C. foetida* | Rhizome | [16] |
| 33 | cimiracemoside D | C_37_H_58_O_11_ | 678.40 | *C. foetida*/  *C. heracleifolia* | Rhizome | [22, 25] |
| 34 | 25-*O*-acetylcimigenol-3-*O*-[3'-*O*-acetyl]-*β*-D-xylopyranoside | C_39_H_60_O_11_ | 704.41 | *C. foetida* | Root | [21] |

Continued

| Number | Chemical constituents | Molecular formula | MW | Source plants | Part of the plants | References |
| --- | --- | --- | --- | --- | --- | --- |
| 35 | 25-*O*-acetylcimigenol-3-*O*-[4'-*O*-acetyl]-*β*-D-xylopyranoside | C_39_H_60_O_11_ | 704.41 | *C. foetida* | Root | [21] |
| 36 | 25-*O*-acetylcimigenol-3-*O*-[2'-*O*- *E* -2-butenoyl]-*β*-D-xylopyranoside | C_41_H_62_O_11_ | 730.00 | *C. foetida* | Root | [21] |
| 37 | 25-*O*-acetylcimigenol-3-*O*-[4'-*O*- *E* -2-butenoyl]-*β*-D-xylopyranoside | C_41_H_62_O_11_ | 730.00 | *C. foetida* | Root | [21] |
| 38 | cimicifoetiside A | C_37_H_58_O_10_ | 662.40 | *C. foetida* | Rhizome | [26] |
| 39 | cimicifoetiside B | C_39_H_60_O_11_ | 704.41 | *C. foetida* | Rhizome | [26] |
| 40 | 20-*O*-acetylcimigenol-3-*O*-*β*-D-xylopyranosyl-3'-*O*-*β*-D-xylopyranoside | C_42_H_66_O_14_ | 794.45 | *C. foetida* | Root | [27] |
| 41 | cimifoside A | C_40_H_64_O_14_ | 768.43 | *C. foetida* | Rhizome | [25] |
| 42 | cimifoside B | C_42_H_66_O_14_ | 794.45 | *C. foetida* | Rhizome | [25] |
| 43 | cimifoetiside A | C_41_H_66_O_14_ | 782.45 | *C. foetida*/  *C. dahurica* | Aerial part | [28] |
| 44 | cimifoetiside B | C_41_H_66_O_14_ | 782.45 | *C. foetida*/  *C. heracleifolia* | Aerial part/  Rhizome | [22, 28] |
| 45 | 25-*O*-acetyl-cimigenol-3-*O*-*β*-D-glc- 1''→2' -*β*-D-xylopyranoside | C_43_H_68_O_15_ | 824.46 | *C. foetida* | Aerial part | [28] |
| 46 | 25-*O*-acetyl-cimigenol-3-*O*-*β*-D-glc- 1''→3' -*β*-D-xylopyranoside | C_43_H_68_O_15_ | 824.46 | *C. foetida* | Aerial part | [28] |
| 47 | cimiside B | C_40_H_64_O_13_ | 752.43 | *C. foetida* | Rhizome | [29] |

Continued

| Number | Chemical constituents | Molecular formula | MW | Source plants | Part of the plants | References |
| --- | --- | --- | --- | --- | --- | --- |
| 48 | 25-dehydrocimigenol | C_30_H_46_O_4_ | 470.34 | *C. dahurica* | Rhizome | [20] |
| 49 | cimifoetiside Ⅲ | C_36_H_56_O_9_ | 632.39 | *C. foetida* | Aerial part | [30] |
| 50 | 25-anhydrocimigenol 3-*O*-*β*-D-xylopyranoside | C_35_H_54_O_8_ | 602.38 | *C. heracleifolia* | Root | [15, 31] |
| 51 | 12-*O*-acetyl-25-anhydrocimicigenol-3-*O*-*β*-D-xylopyranosyl-3'-*O*-*β*-D-xylopyranoside | C_42_H_64_O_14_ | 792.43 | *C. foetida* | Root | [27] |
| 52 | 12*β*-hydroxy-25-anhydrocimigenol | C_30_H_46_O_5_ | 486.33 | *C. foetida* | Aerial part | [32] |
| 53 | actrin-3-one | C_30_H_46_O_5_ | 486.33 | *C. foetida* | Root | [21] |
| 54 | 11-hydroxycomigenol | C_30_H_48_O_6_ | 504.35 | *C. foetida* | Rhizome | [33] |
| 55 | (23*R*,24*S*)-16*β*,23;16*α*,24-diepoxy-cycloartane-3*β*,12*β*,25-triol 3-*O*-*β*-D-xylopyranoside | C_35_H_56_O_9_ | 620.39 | *C. foetida* | Rhizome | [11] |
| 56 | 3-*O*-*β*-D-xylopyranosyl-cimigenol-15-*O*-*β*-D-glucopyranoside | C_41_H_66_O_14_ | 782.45 | *C. dahurica* | Rhizome | [34] |
| 57 | acerinol | C_30_H_46_O_5_ | 486.70 | *C. dahurica* | Rhizome | [6] |
| 58 | 7,8-didehydro-cimigenol | C_30_H_46_O_5_ | 486.68 | *C.foetida*/  *C. dahurica*/  *C. heracleifolia* | Rhizome | [6, 15] |
| 59 | 12*β*-hydroxy-7 8 -en-cimigenol | C_30_H_46_O_6_ | 502.30 | *C. dahurica*/  *C. foetida* | Rhizome | [6, 15] |

Continued

| Number | Chemical constituents | Molecular formula | MW | Source plants | Part of the plants | References |
| --- | --- | --- | --- | --- | --- | --- |
| 60 | 24-*O*-hydroxy-7,8-didehydrohydroshengmanol-3-*O*-*β*-D-xylopyranoside | C_35_H_54_O_9_ | 618.40 | *C. dahurica* | Rhizome | [6] |
| 61 | 25-*O*-acetyl-cimigenol-3-*O*-*β*-D-xylopyranside | C_37_H_56_O_10_ | 660.40 | *C. dahurica* | Rhizome | [6] |
| 62 | (23*R*,24*R*)-16*β*,23;16*α*,24-diepoxy-cycloart-7-en-3*β*,12*β*,15*α*,25-tetraol-3-*O*-*β*-D-xylopyranoside | C_35_H_54_O_10_ | 634.37 | *C. foetida* | Rhizome | [11] |
| 63 | (23*R*,24*R*)-16*β*,23;16*α*,24-diepoxy-12*β*-acetoxy-cycloart-7-en-3*β*,15*α*,25-triol 3-*O*-*β*-D-xylopyranoside | C_37_H_56_O_11_ | 676.38 | *C. foetida* | Rhizome | [11] |
| 64 | 25-*O*-acetylcimigenol-  galactopyranoside | C_38_H_58_O_11_ | 690.40 | *C. foetida* | Aerial part | [30] |
| 65 | cimiricaside A | C_37_H_56_O_10_ | 660.39 | *C. dahurica* | Root | [10] |
| 66 | 2',4'-*O*-diacetyl-24-*epi*-7,8-didehydrocimigenol-3-*O*-xyloside | C_39_H_58_O_11_ | 702.40 | *C. heracleifolia* | Rhizome | [35] |
| 67 | 3'-*O*-acetyl-24-*epi*-7,8-didehydrocimigenol-3-*O*-xyloside | C_37_H_56_O_10_ | 660.39 | *C. heracleifolia* | Rhizome | [35] |
| 68 | 3-keto-24-*epi*-7,8-didehydrocimigenol | C_30_H_44_O_5_ | 484.32 | *C. heracleifolia* | Rhizome | [35] |
| 69 | 24-*epi*-25-*O*-acetylcimigenol | C_32_H_50_O_6_ | 530.36 | *C. foetida* | Rhizome | [16] |

Continued

| Number | Chemical constituents | Molecular formula | MW | Source plants | Part of the plants | References |
| --- | --- | --- | --- | --- | --- | --- |
| 70 | (23*R*,24*R*)-16*β*,23;16*α*,24-diepoxy-cycloartane-3*β*,15*α*,25-triol 3-*O*-*β*-D-xylopyranoside | C_35_H_56_O_9_ | 620.39 | *C. foetida* | Rhizome | [11] |
| 71 | 25-*O*-anhydrocimigenol-3-*O*-*β*-D-xylopyranoside | C_35_H_54_O_8_ | 602.00 | *C. heracleifolia* | Rhizome | [32] |
| 72 | 24-*epi*-cimigenol-3-one | C_30_H_46_O_5_ | 486.33 | *C. foetida*/  *C. heracleifolia* | Rhizome | [16] |
| 73 | heracleifolinol | C_32_H_50_O_7_ | 546.30 | *C. heracleifolia*/  *C. dahurica* | Rhizome | [6] |
| 74 | 24-*epi*-acerinol | C_30_H_46_O_5_ | 486.70 | *C. heracleifolia*/  *C. dahurica/* | Rhizome | [6] |
| 75 | 24-*epi*-25-*O*-acetylacerinol | C_32_H_48_O_6_ | 528.35 | *C. dahurica* | Root | [18] |
| 76 | 24-*O*-acetyl-7,8-didehydrohydroshengmanol-3-*O*-*β*-D- xyloside | C_37_H_58_O_11_ | 678.40 | *C. dahurica* | Rhizome | [6] |
| 77 | 7,8-didehydro-24-acetyldroshengmanol-3-*O*-*α*-L-ara | C_37_H_58_O_11_ | 678.40 | *C. dahurica* | Rhizome | [6] |
| 78 | 7,8-didehydro-24-*O*-acetylhydroshengmanol-3-*O*-xyloside | C_37_H_58_O_11_ | 678.40 | *C. heracleifolia* | Rhizome | [35] |
| 79 | heracleifolinoside D | C_41_H_66_O_15_ | 798.44 | *C. heracleifolia* | Rhizome | [36] |
| 80 | cimifoside C | C_42_H_68_O_15_ | 812.46 | *C. foetida* | Rhizome | [25] |

Continued

| Number | Chemical constituents | Molecular formula | MW | Source plants | Part of the plants | References |
| --- | --- | --- | --- | --- | --- | --- |
| 81 | cimiside C | C_43_H_70_O_16_ | 842.47 | 1. *dahurica/*   *C. heracleifolia* | Rhizome | [36, 37] |
| 82 | 3-xylosyl-24-*O*-acetylhydroshengmanol-15-glucoside | C_43_H_70_O_16_ | 842.47 | *C. dahurica* | Rhizome | [38] |
| 83 | 24-*O*-acetyl-25-anhydroshengmanol-3-*O*-*β*-D-xylopyranoside | C_37_H_58_O_10_ | 662.00 | *C. foetida* | Rhizome | [39] |
| 84 | 24-*O*-acetyl-25-*O*-methyl-7,8-dide-hydrohydroshengmanol-3-*O*-*β*-D-xylopyranoside | C_38_H_60_O_11_ | 692.40 | *C. dahurica* | Rhizome | [6] |
| 85 | 24-*epi*-24-*O*-acetyl-7,8-didehydro-shengmanol-3-*O*-D-galactopyranoside | C_38_H_60_O_12_ | 708.40 | *C. dahurica* | Rhizome | [6] |
| 86 | 24-*epi*-24-*O*-acetyl-7,8-didehydro-shengmanol 3-*O*-*β*-D-xylopyranoside | C_37_H_58_O_11_ | 678.40 | *C. dahurica/*  *C. heracleifolia* | Rhizome | [6, 22] |
| 87 | cimiricaaside E | C_35_H_56_O_10_ | 636.40 | *C. dahurica* | Rhizome | [6] |
| 88 | 24-*epi*-24-*O*-hydroxy-7,8-didehydrosh-engmanol3-*O*-*β*‑D-galactopyranoside | C_36_H_58_O_11_ | 666.40 | *C. dahurica* | Root | [8, 40] |
| 89 | 24-*epi*-24-*O*-hydroxy-7,8-didehydro-shengmanol 3-*O*-*β*‑D-xylopyranoside | C_37_H_58_O_11_ | 678.40 | *C. dahurica* | Root | [40] |
| 90 | heracleifolinoside E | C_41_H_66_O_15_ | 798.44 | *C. heracleifolia* | Rhizome | [36] |

Continued

| Number | Chemical constituents | Molecular formula | MW | Source plants | Part of the plants | References |
| --- | --- | --- | --- | --- | --- | --- |
| 91 | heracleifolinoside F | C_43_H_68_O_16_ | 840.45 | *C. heracleifolia* | Rhizome | [36] |
| 92 | 24-*O*-acetylhydroshengmanol-3-*O*-*β*-D-xyloside | C_37_H_60_O_11_ | 680.41 | *C. foetida* | Rhizome | [16] |
| 93 | 24-*epi*-24-*O*-acetyl-7,8-didehydroshengmanol | C_32_H_50_O_7_ | 546.36 | *C. foetida* | Rhizome | [22, 41] |
| 94 | cimdalglnoside E | C_43_H_68_O_16_ | 840.45 | *C. heracleifolia* | Root | [42] |
| 95 | cimdalglnoside F | C_44_H_70_O_17_ | 870.46 | *C. heracleifolia* | Root | [42] |
| 96 | cimiricaside F | C_40_H_62_O_13_ | 750.42 | *C. heracleifolia* | Root | [10] |
| 97 | heracleifolinoside A | C_41_H_64_O_15_ | 796.42 | *C. heracleifolia* | Rhizome | [36] |
| 98 | heracleifolinoside B | C_41_H_64_O_14_ | 780.43 | *C. heracleifolia* | Rhizome | [36] |
| 99 | heracleifolinoside C | C_41_H_64_O_15_ | 796.42 | *C. heracleifolia* | Rhizome | [36] |
| 100 | 11-dehydro-15*α*-hydroxyximicidol-3-*O*-*β*-D-xylopyranoside | C_35_H_54_O_10_ | 634.37 | *C. foetida* | Root | [12] |
| 101 | cimicifugoside H-2 | C_35_H_54_O_10_ | 634.37 | *C. foetida/*  *C. heracleifolia* | Rhizome | [9, 29] |
| 102 | cimicifugoside H-3 | C_32_H_48_O_9_ | 576.33 | *C. foetida* | Rhizome | [29] |
| 103 | cimicifugoside H-1 | C_35_H_52_O_9_ | 616.36 | *C. foetida* | Rhizome | [29] |
| 104 | cimicifugoside H-5 | C_35_H_52_O_10_ | 632.36 | *C. foetida* | Rhizome | [43] |
| 105 | cimimanol E | C_40_H_60_O_13_ | 748.40 | *C. foetida* | Rhizome | [29] |
| 106 | cimimanol F | C_43_H_62_O_16_ | 834.40 | *C. foetida* | Rhizome | [29] |

Continued

| Number | Chemical constituents | Molecular formula | MW | Source plants | Part of the plants | References |
| --- | --- | --- | --- | --- | --- | --- |
| 107 | (3*β*,12*β*,15*α*,24*R*)-12,2'-diacetoxy-24,25-epoxy-15-hydroxy-16,23-dione- 3-*O*-*α*-L-arabinopyranoside | C_39_H_58_O_12_ | 718.39 | *C. foetida* | Root | [44] |
| 108 | 4',23-*O*-diacetylshengmanol-3-*O*-*α*-L-arabinopyranoside | C_40_H_62_O_10_ | 702.43 | *C. foetida* | Aerial part | [32] |
| 109 | (20*R*,24*R*)-24,25-epoxy-11*β*-hydroxy-7-en-9,19-cyclolanost-3,16,23-trione | C_30_H_42_O_5_ | 482.30 | *C. foetida* | Rhizome | [14] |
| 110 | cimifetidanoside E | C_35_H_52_O_9_ | 616.36 | *C. foetida* | Rhizome | [45] |
| 111 | cimifoetidanol A | C_30_H_44_O_5_ | 484.32 | *C. foetida* | Rhizome | [45] |
| 112 | cimifoetidanol B | C_30_H_44_O_5_ | 484.32 | *C. foetida* | Rhizome | [45] |
| 113 | foetidinoside B | C_41_H_62_O_14_ | 778.41 | *C. foetida* | Rhizome | [46] |
| 114 | cimifetidanoside A | C_35_H_54_O_10_ | 634.37 | *C. foetida* | Rhizome | [45] |
| 115 | cimifetidanoside C | C_35_H_52_O_9_ | 616.36 | *C. foetida* | Rhizome | [45] |
| 116 | cimifetidanoside B | C_35_H_54_O_10_ | 634.37 | *C. foetida* | Rhizome | [45] |
| 117 | cimifetidanoside D | C_35_H_52_O_9_ | 616.36 | *C. foetida* | Rhizome | [45] |
| 118 | cimdalglnoside G | C_42_H_66_O_15_ | 810.44 | *C. dahurica* | Root | [42] |
| 119 | cimdalglnoside H | C_42_H_66_O_15_ | 810.44 | *C. dahurica* | Root | [42] |
| 120 | acetylcimigenol-3-*O*-*α*-L-arabinopyranside | C_37_H_58_O_10_ | 662.85 | *C. foetida* | Aerial part | [23] |
| 121 | 23-acetylshengmanol 3-*O*-*β*-D-xylopyranoside | C_37_H_58_O_10_ | 662.40 | *C. foetida* | Root | [31] |

Continued

| Number | Chemical constituents | Molecular formula | MW | Source plants | Part of the plants | References |
| --- | --- | --- | --- | --- | --- | --- |
| 122 | 4',23-*O*-diacetylshengmanol-3-*O*-*β*-D-xylopyranosyl-3'-*O*-*β*-D-xylopyranoside | C_42_H_66_O_14_ | 794.45 | *C. foetida* | Root | [27] |
| 123 | 23-*O*-acetylshengmanol-3-*O*-*β*-D-glc-(1''→3')-β-D-xylopyranoside | C_43_H_68_O_15_ | 824.46 | *C. foetida* | Aerial part | [28] |
| 124 | 23-*O*-acetylshengmanol-3-*O*-(2'-*O*-malonyl)-xylopyranoside | C_40_H_60_O_13_ | 748.40 | *C. dahurica* | Rhizome | [47] |
| 125 | 23-*O*-acetyl-7,8-didehydroshengmanol | C_32_H_48_O_6_ | 528.35 | *C. dahurica* | Rhizome | [41] |
| 126 | 23-*O*-acetyl-7,8-didehydroshengmanol-3-*O*-*α*-arabinopyranoside | C_37_H_56_O_10_ | 660.39 | *C. dahurica/*  *C. heracleifolia* | Rhizome | [8, 41] |
| 127 | 23-*O*-acetyl-7,8-didehydroshengmanol-3-xylopyranoside | C_37_H_56_O_10_ | 660.39 | *C. dahurica* | Rhizome | [8, 47] |
| 128 | 23-*O*-acetyl-7,8-didehydroshengmanol-3-*O*-(2'-*O*-malonyl)-xylopyranoside | C_40_H_58_O_13_ | 746.39 | *C. dahurica* | Rhizome | [47] |
| 129 | cimdahxynoside A | C_43_H_68_O_15_ | 824.46 | *C. dahurica* | Root | [17] |
| 130 | cimdahxynoside B | C_43_H_68_O_15_ | 824.46 | *C. dahurica* | Root | [17] |
| 131 | cimdahxynoside C | C_43_H_66_O_15_ | 822.44 | *C. dahurica* | Root | [17] |
| 132 | cimiacerin B | C_30_H_48_O_5_ | 488.35 | *C. foetida/*  *C. dahurica* | Rhizome | [8, 16] |
| 133 | 12*β*-hydroxycimiacerol | C_30_H_48_O_6_ | 504.35 | *C. foetida* | Rhizome | [48] |
| 134 | 12*β*-*O*-acetylcimiracemonol | C_31_H_48_O_7_ | 532.34 | *C. foetida* | Aerial part | [49] |

Continued

| Number | Chemical constituents | Molecular formula | MW | Source plants | Part of the plants | References |
| --- | --- | --- | --- | --- | --- | --- |
| 135 | cimifetidanoside H | C_37_H_58_O_11_ | 678.40 | *C. foetida* | Rhizome | [45] |
| 136 | cimiaceroside B | C_35_H_56_O_9_ | 620.39 | *C. foetida* | Rhizome | [50] |
| 137 | cimiaceroside C | C_40_H_64_O_13_ | 752.43 | *C. foetida* | Rhizome | [25] |
| 138 | 23-methoxy-24-aminocimiacerol-3-*O*-*β*-D-xylopyranoside | C_36_H_59_NO_8_ | 633.42 | *C. foetida* | Rhizome | [48] |
| 139 | cimiaceroside E | C_40_H_62_O_11_ | 718.43 | *C. foetida* | Rhizome | [50] |
| 140 | cimiacerol-1(2)-en-3-one | C_30_H_44_O_5_ | 484.32 | *C. foetida* | Root | [27] |
| 141 | 12*β*-hydroxy-7(8)-en-cimiacerol-3-*O*-*α*-L-arabinopyranoside | C_35_H_54_O_10_ | 634.81 | *C. foetida* | Root | [48] |
| 142 | 12*β*-hydroxy-7(8)-en-cimiacerol-3-*O*-*β*-D-xylopyranoside | C_35_H_54_O_10_ | 634.81 | *C. foetida* | Root | [48] |
| 143 | cimdalglnoside A | C_57_H_84_O_22_ | 1120.55 | *C. dahurica* | Root | [42] |
| 144 | cimdalglnoside B | C_63_H_94_O_27_ | 1282.60 | *C. dahurica* | Root | [42] |
| 145 | cimdalglnoside C | C_58_H_86_O_23_ | 1150.56 | *C. dahurica* | Root | [42] |
| 146 | cimdalglnoside D | C_56_H_82_O_22_ | 1106.53 | *C. dahurica* | Root | [42] |
| 147 | 20*S*,22*R*,23*S*,24*R*-16*β*,23;22,25-diepoxy-cycloartane-3*β*,23,24-triol-3-O-(6-O-*trans*-isoferuloy-*β*-D-glucopyranosyl)-(1-2)-*β*-D-glucopyranosyl-(1-2)-*β*-D-xylopyranoside | C_57_H_84_O_22_ | 1120.55 | *C. dahurica* | Root | [42] |
| 148 | actein | C_37_H_56_O_11_ | 676.8 | *C. foetida*/  *C. dahurica*/  *C. heracleifolia* | Rhizome | [9, 22, 45] |
| 149 | 26-deoxyactein | C_37_H_56_O_10_ | 660.39 | *C. foetida* | Rhizome | [50] |

Continued

| Number | Chemical constituents | Molecular formula | MW | Source plants | Part of the plants | References |
| --- | --- | --- | --- | --- | --- | --- |
| 150 | acteol-3-*O*-*β*-D-xylopyranosyl-3'-*O*-*β*-D-xylopyranoside | C_42_H_64_O_15_ | 808.42 | *C. foetida* | Root | [27] |
| 151 | 23-*epi*-26-deoxyactein | C_37_H_56_O_10_ | 660.39 | *C. foetida/*  *C. dahurica* | Rhizome | [45] |
| 152 | cimiracemoside Ⅰ | C_35_H_52_O_8_ | 600.40 | *C. dahurica* | Rhizome | [6] |
| 153 | 7,8-didehydro-26-deoxyactein | C_37_H_54_O_10_ | 658.37 | *C. foetida* | Rhizome | [50] |
| 154 | 26-deoxyacetylacteol-7(8)-en-3-*O*-*β*-D-xylopyranosyl-3'-*O*-*β*-D-xylopyranoside | C_42_H_62_O_14_ | 790.41 | *C. foetida* | Root | [27] |
| 155 | 3'-*O*-acetylactein | C_39_H_58_O_12_ | 718.39 | *C. foetida* | Root | [21] |
| 156 | acteinol | C_32_H_48_O_7_ | 544.34 | *C. foetida* | Root | [21] |
| 157 | 26-deoxyacteinol | C_32_H_48_O_6_ | 528.35 | *C. foetida* | Root | [21] |
| 158 | 3'-*O*-acetyl-23-*epi*-26-deoxyactein | C_39_H_58_O_11_ | 702.40 | *C. foetida* | Root | [21] |
| 159 | cimifoside D | C_42_H_64_O_14_ | 792.43 | *C. foetida* | Rhizome | [25] |
| 160 | foetidinol | C_27_H_40_O_5_ | 444.00 | *C. foetida* | Rhizome | [29] |
| 161 | foetidinol-3-*O*-β-xyloside | C_32_H_48_O_9_ | 576.33 | *C. foetida* | Rhizome | [51] |
| 162 | 3*β*,15*α*,16*α*,24*α*-tetrahydroxy-25,26,27-trinor-16,24-cyclo-cycloartane-7-en-23-one 3-*O*-*β*-D-xylopyranoside | C_32_H_48_O_9_ | 576.33 | *C. heracleifolia* | Rhizome | [52] |
| 163 | 12*β*-acetoxy-3*β*,15*α*,16*α*,24*α*-tetrahydroxy-25,26,27-trinor-16,24-cyclo-cycloart-7-en-23-one 3-*O*-*β*-D-xylopyranoside | C_34_H_54_O_11_ | 634.34 | *C. heracleifolia* | Rhizome | [52] |

Continued

| Number | Chemical constituents | Molecular formula | MW | Source plants | Part of the plants | References |
| --- | --- | --- | --- | --- | --- | --- |
| 164 | cimicifugoside H-6 | C_32_H_48_O_10_ | 592.32 | *C. foetida* | Rhizome | [43] |
| 165 | 3*β*,15*α*,16*α*,24*α*-tetrahydroxy-25,26,27-trinor-16,24-cyclo-cycloartane-23-one 3-*O*-*β*-D-xylopyranoside | C_32_H_50_O_9_ | 578.35 | *C. heracleifolia* | Rhizome | [52] |
| 166 | 12*β*-acetoxy-7(8)-en-cimilactone-3-*O*-*α*-L-arabinopyranoside | C_33_H_48_O_9_ | 588.33 | *C. foetida* | Rhizome | [48] |
| 167 | cimilactone B | C_33_H_48_O_9_ | 588.33 | *C. dahurica* | Aerial part | [53] |
| 168 | 12*β*-hydroxy-7(8)-en-cimilactone-3-*O*-*α*-L-arabinopyranoside | C_31_H_46_O_8_ | 546.32 | *C. foetida* | Root | [48] |
| 169 | 3*β*,11*β*-dihydroxy-24,25,26,27-tetranor-cycloart-7-en-23,16*β*-olide 3-*O*-*β*-D-xylopyranoside | C_31_H_46_O_8_ | 546.32 | *C. heracleifolia* | Rhizome | [52] |
| 170 | cimilactone C | C_28_H_42_O_5_ | 458.30 | *C. dahurica* | Aerial part | [54] |
| 171 | cimilactone A | C_33_H _50_O_9_ | 590.35 | *C. dahurica/*  *C. foetida* | Aerial part | [15, 53] |
| 172 | 12*β*-acetoxy-cimilactone-3-*O*-*α*-L- arabinopyranoside | C_33_H_50_O_9_ | 590.35 | *C. foetida* | Root | [48] |
| 173 | cimilactone E | C_35_H_52_O_10_ | 632.36 | *C. dahurica/*  *C. foetida* | Root | [15, 55] |
| 174 | cimilactone F | C_37_H_54_O_10_ | 658.37 | *C. dahurica/*  *C. foetida* | Root | [15, 55] |

Continued

| Number | Chemical constituents | Molecular formula | MW | Source plants | Part of the plants | References |
| --- | --- | --- | --- | --- | --- | --- |
| 175 | cimilactone K | C_38_H_58_O_13_ | 722.39 | *C. foetida/*  *C. heracleifolia* | Root | [22, 27] |
| 176 | (20*S*,24*R*)-20,24-epoxy-15*α*,23*β*,25-trihydroxy-3-(*β*-D-xylopyranosyloxy)-9,19-cycloart-7-en-16-one | C_35_H_54_O_10_ | 634.37 | *C. foetida* | Root | [12] |
| 177 | *β*-sitosterol | C_29_H_50_O | 414.72 | *C. foetida* | Rhizome | [9, 32] |
| 178 | daucosterol | C_35_H_62_O_6_ | 590.45 | *C. foetida* | Root/Rhizome | [9, 56] |
| 179 | 12*β*-acetylcimigenol-3-*O*-*β*-D-xylopyranoside | C_37_H_58_O_10_ | 662.40 | *C. foetida* | Rhizome | [45] |
| 180 | foetinoside | C_35_H_60_O_9_ | 624.42 | *C. foetida* | Rhizome | [16] |
| 181 | 23,24-diacetoxy-3,15,25-trihydroxy-cycloart-7-en-16-one-3-*O*-xylopyranoside | C_39_H_60_O_12_ | 720.41 | *C. heracleifolia* | Rhizome | [52] |
| 182 | (24*R*)-3,15,25-trihydroxy-cycloart-7-en-16-one-3-*O*-xylopyranoside | C_37_H_58_O_11_ | 678.40 | *C. heracleifolia/*  *C. dahurica* | Rhizome | [8, 52] |
| 183 | cimisterol A | C_29_H_46_O_3_ | 442.34 | *C. foetida* | Rhizome | [49] |
| 184 | 12*β*-hydroxycimigenol-3-*O*-*β*-D-xylopyranoside | C_35_H_56_O_9_ | 620.39 | *C. foetida* | Rhizome | [45] |
| 185 | rubraside A | C_35_H_56_O_9_ | 620.49 | *C. dahurica* | Rhizome | [57] |

Continued

| Number | Chemical constituents | Molecular formula | MW | Source plants | Part of the plants | References |
| --- | --- | --- | --- | --- | --- | --- |
| 186 | 25,3'-*O*-diacetylcimigenol-3-*β*-D-xylopyranoside | C_35_H_56_O_9_ | 620.39 | *C. foetida* | Rhizome | [45] |
| 187 | cimiracemoside E | C_37_H_58_O_11_ | 678.80 | *C. dahurica* | Rhizome | [57] |
| 188 | aceriphyllic A | C_30_H_48_O_4_ | 472.36 | *C. foetida* | Aerial part | [49] |
| 189 | 3*β*,6*β*-dihydroxyolean-12-en-27-oic acid | C_30_H_48_O_4_ | 472.36 | *C. foetida* | Aerial part | [49] |
| 190 | 24-acetoxy-15,16-seco-cycloar-tane 3-*O*-xylopyranoside | C_37_H_58_O_12_ | 694.39 | *C. foetida* | Rhizome | [58] |
| 191 | (24*R*)-15,16-seco-cycloar-tane 3-*O*-xylopyranoside | C_35_H_56_O_11_ | 652.38 | *C. foetida* | Rhizome | [58] |
| 192 | (24*S*)-15,16-seco-cycloar-tane 3-*O*-xylopyranoside | C_35_H_56_O_11_ | 652.38 | *C. foetida* | Rhizome | [58] |
| 193 | 25,4'-*O*-diacetylcimigenol-3*β*-D-xylopyranoside | C_39_H_60_O_10_ | 688.42 | *C. foetida* | Rhizome | [45] |
| 194 | cimdahxynoside J | C_43_H_64_O_16_ | 836.42 | *C. dahurica* | Root | [17] |
| 195 | cimiricaside D | C_35_H_52_O_10_ | 632.36 | *C. dahurica* | Root | [10] |
| 196 | yunnanterpene G | C_30_H_44_O_6_ | 500.31 | *C. foetida* | Root | [59] |
| 197 | cimimanol A | C_38_H_58_O_12_ | 706.39 | *C. foetida* | Rhizome | [29] |
| 198 | cimimanol B | C_35_H_56_O_10_ | 636.39 | *C. foetida* | Rhizome | [29] |
| 199 | cimimanol C | C_42_H_66_O_14_ | 794.45 | *C. foetida* | Rhizome | [29] |

Continued

| Number | Chemical constituents | Molecular formula | MW | Source plants | Part of the plants | References |
| --- | --- | --- | --- | --- | --- | --- |
| 200 | (16*S*,20*S*,24*R*)-12*β*-acetoxy-16,23-epoxy-24,25-dihydroxy-3*β*-(*β*-D-xylopyranosyloxy)-9,19-cyclolanost-22(23)-ene | C_37_H_58_O_10_ | 662.40 | *C. foetida* | Rhizome | [29] |
| 201 | foetidinoside A | C_41_H_70_O_13_ | 770.48 | *C. foetida* | Rhizome | [46] |
| 202 | foetidinoside C | C_41_H_70_O_14_ | 786.48 | *C. foetida/*  *C. heracleifolia* | Rhizome | [22, 46] |
| 203 | foetidinoside D | C_41_H_70_O_14_ | 786.48 | *C. foetida* | Rhizome | [46] |
| 204 | foetidinoside E | C_41_H_70_O_15_ | 802.47 | *C. foetida/*  *C. dahurica* | Rhizome | [46] |
| 205 | cimdahxynoside F | C_35_H_54_O_9_ | 618.38 | *C. dahurica* | Root | [17] |
| 206 | cimdahxynoside G | C_35_H_52_O_8_ | 600.37 | *C. dahurica* | Root | [17] |
| 207 | cimdahxynoside H | C_35_H_54_O_8_ | 602.38 | *C. dahurica* | Root | [17] |
| 208 | cimdahxynoside I | C_41_H_66_O_14_ | 782.45 | *C. dahurica* | Root | [17] |
| 209 | 2',23-*O*-diacetylshengmanol-3-*O*-*α*-L-arabinopyranoside. | C_39_H_60_O_11_ | 704.41 | *C. dahurica* | Aerial part | [32] |
| 210 | 2',24-*O*-diacetylisodahurinol-3-*O*-*α*-L-arabinopyranoside | C_39_H_60_O_11_ | 704.41 | *C. dahurica* | Aerial part | [32] |
| 211 | 24-*O*-acetylisodahurinol-3-*O*-*α*-L-arabinopyranoside | C_37_H_58_O_10_ | 662.40 | *C. dahurica* | Aerial part | [32] |

Continued

| Number | Chemical constituents | Molecular formula | MW | Source plants | Part of the plants | References |
| --- | --- | --- | --- | --- | --- | --- |
| **Phenylpropanoids** | | | | | | |
| 212 | 4-*O*-acetyl caffeic acid | C_11_H_10_O_5_ | 222.00 | *C. foetida* | Rhizome | [60] |
| 213 | caffeic acid | C_9_H_8_O_4_ | 180.00 | *C. foetida*/  *C. dahurica*/  *C. heracleifolia* | Rhizome | [60, 61] |
| 214 | isoferulic acid | C_10_H_10_O_4_ | 194.18 | *C. foetida*/  *C. dahurica*/  *C. heracleifolia* | Rhizome | [9, 60] |
| 215 | ferulic acid | C_10_H_10_O_4_ | 194.18 | *C. foetida*/  *C. dahurica*/  *C. heracleifolia* | Rhizome | [60, 61] |
| 216 | 3-methoxyl-4-ethyoxyl-phenylpropionic acid | C_12_H_14_O_4_ | 222.09 | *C. dahurica* | Rhizome | [20] |
| 217 | *trans*-isoferulic acid 3-*O*-*β*-D-allopyranoside | C_16_H_20_O_9_ | 356.11 | *C. dahurica* | Rhizome | [8, 62] |
| 218 | *trans*-caffeic acid 4-*O*-*β*-D-allopyranoside | C_15_H_18_O_9_ | 342.10 | *C. dahurica* | Rhizome | [62] |
| 219 | cinnamic acid | C_9_H_8_O_2_ | 148.16 | *C. dahurica* | Rhizome | [57] |
| 220 | p-hydroxy cinnamic acid | C_9_H_8_O_3_ | 164.16 | *C. dahurica* | Rhizome | [57] |
| 221 | 3,4-dimethoxycinnamic acid | C_11_H_12_O_4_ | 208.07 | *C. dahurica* | Rhizome | [63] |
| 222 | caffeic acid 3-*O*-*β*-D-glucopyranoside | C_15_H_18_O_9_ | 342.10 | *C. dahurica* | Rhizome | [8, 64] |

Continued

| Number | Chemical constituents | Molecular formula | MW | Source plants | Part of the plants | References |
| --- | --- | --- | --- | --- | --- | --- |
| 223 | *trans*-ferulic acid-4-*O*-*β*-D-galaopyranoside | C_16_H_20_O_9_ | 356.11 | *C. dahurica* | Rhizome | [63] |
| 224 | methyl caffeate | C_10_H_10_O_4_ | 194.18 | *C. dahurica* | Rhizome | [57] |
| 225 | ethyl caffeate | C_11_H_12_O_4_ | 208.21 | *C. dahurica* | Rhizome | [57] |
| 226 | ethyl ferulate | C_12_H_14_O_4_ | 222.09 | *C. dahurica* | Rhizome | [64] |
| 227 | caffeic ester glucoside | C_15_H_18_O_9_ | 342.10 | *C. dahurica* | Rhizome | [64] |
| 228 | 1-*O*-feruloyl-*β*-D-glucopyranoside | C_16_H_20_O_9_ | 356.11 | *C. dahurica* | Rhizome | [63] |
| 229 | sinapic acid | C_11_H_12_ O_5_ | 224.21 | *C. foetida* | Rhizome | [60] |
| 230 | ferulic acid methyl ester | C_11_H_12_O_4_ | 208.07 | *C. dahurica* | Root | [65] |
| 231 | (*E*)-sinapic acid 4-*O*-*β*-D-glucoside | C_17_H_22_O_10_ | 386.12 | *C. dahurica* | Rhizome | [8, 66] |
| 232 | fukiic acid | C_11_H_12_O_8_ | 272.05 | *C. dahurica*/  *C. heracleifolia* | Rhizome | [8, 67] |
| 233 | piscidic acid | C_11_H_12_O_7_ | 256.06 | *C. foetida*/  *C. dahurica*/  *C. heracleifolia* | Rhizome | [61, 67] |
| 234 | 4-hydroxy-2-(2*E*-8-hydroxy-3,7-dimethyl-2-octenyl)-5-acetic acid *β*-D-glucopyranoside | C_24_H_36_O_10_ | 484.23 | *C. dahurica* | Rhizome | [63] |
| 235 | cimicifugic acid | C_20_H_20_O_7_ | 372.37 | *C. foetida* | Rhizome | [60] |

Continued

| Number | Chemical constituents | Molecular formula | MW | Source plants | Part of the plants | References |
| --- | --- | --- | --- | --- | --- | --- |
| 236 | carboxymethyl isoferulate | C_12_H_12_O_6_ | 252.06 | *C. dahurica* | Rhizome | [63] |
| 237 | 4'-methoxyl-3'-hydroxy-carboxy-benzoyl isoferulic acid anhydride | C_18_H_16_O_7_ | 344.09 | *C. dahurica/*  *C. heracleifolia* | Rhizome | [22, 63] |
| 238 | cimicifugaside F | C_26_H_30_O_12_ | 534.17 | *C. dahurica* | Rhizome | [66] |
| 239 | (+)(2*S*,3*R*)-2-(4-hydroxy-3-methoxyphenyl)-3-[(*β*-D-glucopyranosyloxy)methyl]-7-methoxybenzofuran-5-propenoic acid | C_26_H_30_O_12_ | 534.17 | *C. dahurica* | Rhizome | [66] |
| 240 | esculetin | C_9_H_6_O_4_ | 178.14 | *C. foetida* | Rhizome | [60] |
| 241 | cimiciphenone | C_18_H_16_O_7_ | 344.09 | *C. dahurica* | Root | [68] |
| 242 | cimiracemate A | C_18_H_16_O_7_ | 344.09 | *C. dahurica* | Root | [68] |
| 243 | 3,5-di-*O*-caffeoylquinic acid | C_27_H_28_O_10_ | 512.17 | *C. dahurica* | Root | [68] |
| 244 | cimicifugic G | C_22_H_22_O_11_ | 462.12 | *C. dahurica* | Rhizome | [64] |
| 245 | cimicifugic acid D | C_20_H_18_O_10_ | 418.09 | *C. dahurica*/  *C. heracleifolia* | Rhizome | [22, 69] |
| 246 | cimicifugic acid A | C_21_H_20_O_11_ | 448.10 | *C. foetida*/  *C. dahurica*/  *C. heracleifolia* | Rhizome | [61, 69] |
| 247 | cimicifugic B | C_21_H_20_O_11_ | 448.10 | *C. dahurica* | Rhizome | [64] |
| 248 | cimicifugic E | C_21_H_20_O_10_ | 432.11 | *C. dahurica* | Rhizome | [64] |

Continued

| Number | Chemical constituents | Molecular formula | MW | Source plants | Part of the plants | References |
| --- | --- | --- | --- | --- | --- | --- |
| 249 | cimicifugic acid F | C_21_H_20_O_10_ | 432.11 | *C. dahurica*/  *C. heracleifolia* | Rhizome | [22, 69] |
| 250 | fukinolic acid | C_20_H_18_O_11_ | 434.08 | *C. foetida*/  *C. dahurica*/  *C. heracleifolia* | Root/Rhizome | [61, 70] |
| 251 | cimicifugic acid L | C_22_H_22_O_10_ | 466.12 | *C. dahurica* | Rhizome | [69] |
| 252 | shomaside B | C_27_H_30_O_15_ | 594.16 | *C. heracleifolia*/  *C. dahurica* | Rhizome | [67] |
| 253 | shomaside A | C_27_H_30_O_16_ | 610.15 | *C. heracleifolia*/  *C. dahurica* | Rhizome | [22, 64] |
| 254 | 2-isoferuloyl piscidic acid | C_21_H_20_O_10_ | 432.11 | *C. dahurica* | Rhizome | [63] |
| 255 | 2-feruloyl piscidic acid | C_21_H_20_O_10_ | 432.11 | C. *foetida/*  *C. dahurica* | Rhizome | [61, 63] |
| 256 | cimicifugaside C | C_27_H_34_O_13_ | 566.20 | *C. dahurica* | Rhizome | [69] |
| 257 | cimicifugaside D | C_27_H_34_O_13_ | 566.20 | *C. dahurica* | Rhizome | [69] |
| 258 | cimicifugaside E | C_27_H_36_O_13_ | 568.22 | *C. dahurica* | Rhizome | [69] |
| 259 | cimicifugaside A | C_32_H_38_O_17_ | 694.21 | *C. dahurica* | Rhizome | [69] |
| 260 | cimicifugaside B | C_33_H_40_O_18_ | 724.22 | *C. dahurica* | Rhizome | [69] |
| 261 | cimicifugamide B | C_24_H_29_NO_10_ | 491.18 | *C. dahurica* | Rhizome | [69] |
| 262 | cimicifugamide C | C_25_H_31_NO_10_ | 505.19 | *C. dahurica* | Rhizome | [69] |
| 263 | cimicifugamide D | C_24_H_29_NO_8_ | 459.19 | *C. dahurica* | Rhizome | [69] |

Continued

| Number | Chemical constituents | Molecular formula | MW | Source plants | Part of the plants | References |
| --- | --- | --- | --- | --- | --- | --- |
| 264 | *trans*-N-feruloyl-3',4'- dihydroxyphenylethylamine | C_18_H_19_NO_5_ | 329.13 | *C. dahurica* | Rhizome | [62] |
| 265 | *trans*-N-feruloyltyramine | C_18_H_19_NO_4_ | 313.3 | *C. dahurica* | Rhizome | [62] |
| 266 | *trans*-feruloyltyramine 4-*O*-*β*-D-allopyranoside | C_24_H_29_NO_9_ | 475.18 | *C. heracleifolia* | Rhizome | [67] |
| 267 | *trans*-Feruloyl-(3-*O*-methyl) dopamine 4-*O*-*β*-D-allopyranoside | C_25_H_31_NO_10_ | 505.19 | *C. heracleifolia* | Rhizome | [67] |
| 268 | (2*E*)-3-[4-(*β*-D-allopyranosyl)-3-methoxy-phenyl]-N-[2-(4-hydroxy-3-methoxyphenyl) ethyl]-2-propenamide | C_25_H_31_NO_10_ | 505.19 | *C. dahurica* | Rhizome | [34] |
| 269 | *N*-*trans*-3'-methoxy-4'-feruloyl-tyramine-4-*O*-*β*-D-glucoside | C_25_H_31_NO_10_ | 505.00 | *C. dahurica* | Rhizome | [66] |
| 270 | cimicifugamide | C_25_H_31_O_10_N | 505.19 | *C. dahurica/*  *C. heracleifolia* | Rhizome | [8, 71] |
| 271 | (+)-pinoresinol di-*O*-*β*-D-allopyranoside | C_32_H_42_O_16_ | 682.25 | *C. dahurica* | Rhizome | [34] |
| 272 | (－)-syringaresinol-4-*O*-*β*-D-alloside | C_28_H_36_O_13_ | 580.22 | *C. dahurica* | Rhizome | [62] |
| 273 | actaealactone | C_18_H_14_O_8_ | 358.07 | *C. dahurica* | Root/Rhizome | [70] |
| 274 | (－)-syringaresinol | C_20_H_26_O_6_ | 362.17 | *C. dahurica* | Rhizome | [66] |

Continued

| Number | Chemical constituents | Molecular formula | MW | Source plants | Part of the plants | References |
| --- | --- | --- | --- | --- | --- | --- |
| 275 | (+)-isolarisiresinol 3-*O*-*β*-D-glucoside | C_26_H_34_O_11_ | 522.21 | *C. dahurica* | Rhizome | [66] |
| 276 | syringaresinol di-*O*-*β*-D-allopyranoside | C_34_H_46_O_18_ | 742.27 | *C. dahurica* | Rhizome | [34] |
| 277 | shomaside H | C_28_H_32_O_15_ | 608.17 | *C. dahurica* | Rhizome | [62] |
| 278 | 6, 6'-di-*O*-sinapoylsurcose | C_34_H_42_O_19_ | 754.23 | *C. dahurica* | Rhizome | [16] |
| **Chromones** | | | | | | |
| 279 | peucenin | C_15_H_16_O_4_ | 260.10 | *C. dahurica* | Rhizome | [20] |
| 280 | kellol | C_13_H_10_O_5_ | 246.05 | *C. dahurica* | Rhizome | [16] |
| 281 | norkhellol | C_12_H_8_O_5_ | 232.04 | C. *foetida* | Rhizome | [16] |
| 282 | 6'-hydroxylangelicain | C_15_H_16_O_7_ | 308.28 | C. *foetida* | Rhizome | [9] |
| 283 | cimifugin | C_16_H_18_O_6_ | 306.31 | C. *foetida/*  *C. dahurica* | Rhizome | [9, 72] |
| 284 | norcimifugin | C_15_H_16_O_6_ | 292.09 | C. *foetida* | Rhizome | [16] |
| 285 | prim-*O*-glucosylcimifugin | C_22_H_28_O_11_ | 468.45 | *C. dahurica* | Rhizome | [34] |
| 286 | prim-*O*-glucosylangelicain | C_21_H_26_O_11_ | 454.15 | C. *foetida* | Rhizome | [9] |
| 287 | norkhelloside | C_23_H_26_O_14_ | 526.13 | *C. heracleifolia* | Rhizome | [36] |
| 288 | cimifugin-4'-*O*-[6''-feruloyl]-*β*-D-glucopyranoside | C_32_H_36_O_14_ | 644.21 | C. *foetida* | Rhizome | [16] |
| **Alkalodis** | | | | | | |
| 289 | (*E*)-3- 3'-methyl-2'-butenylidene -2-indolinone | C_13_H_13_NO | 199.10 | *C. dahurica* | Root | [68] |

Continued

| Number | Chemical constituents | Molecular formula | MW | Source plants | Part of the plants | References |
| --- | --- | --- | --- | --- | --- | --- |
| 290 | (*E*)-3- 3'-methyl-2'-butenylidene -1-methyl-2-indolinone | C_14_H_15_NO | 213.12 | *C. dahurica* | Root | [68] |
| 291 | (*Z*)-3- 3'-methyl-2'-butenylidene -1-methyl-2-indolinone | C_14_H_15_NO | 213.12 | *C. dahurica* | Root | [68] |
| 292 | (*E*)-3-(3′-methylbutylidene)-2-indolinone | C_13_H_15_NO | 201.12 | *C. foetida* | Rhizome | [16] |
| 293 | 3-(3-methyl-1-oxo-2-butenyl) 1H indole | C_13_H_13_NO | 199.10 | *C. foetida* | Rhizome | [16] |
| 294 | imicifoetone A | C_26_H_22_N_2_O_2_ | 394.17 | *C. foetida* | Rhizome | [73] |
| 295 | imicifoetone B | C_31_H_30_N_2_O_2_ | 462.23 | *C. foetida* | Rhizome | [73] |
| **Terpenoids** | | | | | | |
| 296 | cimicifugolide A | C_10_H_14_O_4_ | 198.09 | *C. foetida* | Rhizome | [74] |
| 297 | cimicifugolide B | C_11_H_18_O_5_ | 230.12 | *C. foetida* | Rhizome | [74] |
| 298 | cimicifugolide C | C_10_H_12_O_3_ | 180.08 | *C. foetida* | Rhizome | [74] |
| 299 | (3*S*)-4*α*-hydroxy-3-(2-hydroxyethylidene)-5*β*-(2-methylprop-1-enyl) dihydrofuran-2-one | C_10_H_14_O_4_ | 198.09 | *C. foetida* | Rhizome | [74] |
| 300 | 3-hydroxymegastigmasta-5, 7-dien-9-one-3-*O*-*β*-D-glucopyranoside | C_19_H_32_O_7_ | 372.21 | *C. dahurica* | Aerial part | [72] |
| 301 | picrionoside A | C_19_H_30_O_7_ | 370.20 | *C. dahurica* | Aerial part | [72] |
| 302 | icariside B_2_ | C_19_H_30_O_8_ | 386.19 | *C. dahurica* | Aerial part | [72] |
| 303 | citroside A | C_19_H_30_O_8_ | 386.19 | *C. dahurica* | Aerial part | [72] |

Continued

| Number | Chemical constituents | Molecular formula | MW | Source plants | Part of the plants | References |
| --- | --- | --- | --- | --- | --- | --- |
| 304 | paeoniflorin | C_23_H_28_O_11_ | 480.16 | *C. dahurica* | Rhizome | [34] |
| 305 | geniposide | C_17_H_24_O_10_ | 388.37 | *C. dahurica* | Rhizome | [34] |
| **Others** | | | | | | |
| 306 | cimidahurine | C_14_H_20_O_8_ | 316.12 | *C. dahurica/*  *C. heracleifolia* | Aerial part | [8, 72] |
| 307 | 3,5-dihydroxyphenethanol 3-*O*-*β*-D-allopyranoside | C_14_H_20_O_8_ | 316.12 | *C. dahurica* | Rhizome | [62] |
| 308 | cimidahurinine | C_14_H_20_O_8_ | 316.12 | *C. foetida*/  *C. dahurica*/  *C. heracleifolia* | Aerial part/  Rhizome | [61, 72] |
| 309 | benzoic acid 4-*O*-*β*-D-glucoside | C_13_H_16_O_8_ | 300.08 | *C. dahurica* | Rhizome | [66] |
| 310 | 4-hydroxyphenylacetic acid | C_8_H_8_O_3_ | 152.15 | *C. dahurica* | Rhizome | [57] |
| 311 | 5-hydroxy-2-methoxybenzoic acid | C_8_H_8_O_4_ | 168.04 | *C. dahurica* | Rhizome | [66] |
| 312 | alopecuquinone | C_14_H_18_O_8_ | 314.10 | *C. dahurica* | Aerial part | [72] |
| 313 | quercetin 3-*O*-*β*-D-galactopyranoside | C_21_H_20_O_12_ | 464.10 | *C. dahurica* | Aerial part | [72] |
| 314 | kaempferol 3-*O*-*β*-D-galactopyranoside | C_21_H_20_O_11_ | 448.10 | *C. dahurica* | Aerial part | [72] |
| 315 | sucrose | C_12_H_22_O_11_ | 342.30 | C. *heracleifolia* | Rhizome | [75] |
| 316 | [*β*-D-Fru*f*-(2→1)]_2_-*α*-D-Glc*p* | C_18_H_32_O_16_ | 504.17 | C. *heracleifolia* | Rhizome | [75] |

Continued

| Number | Chemical constituents | Molecular formula | MW | Source plants | Part of the plants | References |
| --- | --- | --- | --- | --- | --- | --- |
| 317 | [*β*-D-Fru*f*-(2→1)]_3_-*α*-D-Glc*p* | C_24_H_42_O_21_ | 666.22 | *C. heracleifolia* | Rhizome | [75] |
| 318 | [*β*-D-Fru*f*-(2→1)]_4_-*α*-D-Glc*p* | C_30_H_52_O_26_ | 828.28 | *C. heracleifolia* | Rhizome | [75] |
| 319 | [*β*-D-Fru*f*-(2→1)]_5_-*α*-D-Glc*p* | C_36_H_62_O_31_ | 990.33 | *C. heracleifolia* | Rhizome | [75] |
| 320 | [*β*-D-Fru*f*-(2→1)]_6_-*α*-D-Glc*p* | C_42_H_72_O_36_ | 1152.38 | *C. heracleifolia* | Rhizome | [75] |
| 321 | [*β*-D-Fru*f*-(2→1)]_7_-*α*-D-Glc*p* | C_48_H_82_O_41_ | 1314.43 | *C. heracleifolia* | Rhizome | [75] |
| 322 | [*β*-D-Fru*f*-(2→1)]_8_-*α*-D-Glc*p* | C_54_H_92_O_46_ | 1476.49 | *C. heracleifolia* | Rhizome | [75] |
| 323 | [*β*-D-Fru*f*-(2→1)]_9_-*α*-D-Glc*p* | C_60_H_102_O_51_ | 1638.54 | *C. heracleifolia* | Rhizome | [75] |
| 324 | [*β*-D-Fru*f*-(2→1)]_10_-*α*-D-Glc*p* | C_66_H_112_O_56_ | 1800.59 | *C. heracleifolia* | Rhizome | [75] |
| 325 | [*β*-D-Fru*f*-(2→1)]_11_-*α*-D-Glc*p* | C_72_H_122_O_61_ | 1962.65 | *C. heracleifolia* | Rhizome | [75] |
| 326 | [*β*-D-Fru*f*-(2→1)]_12_-*α*-D-Glc*p* | C_78_H_132_O_66_ | 2124.70 | *C. heracleifolia* | Rhizome | [75] |
| 327 | [*β*-D-Fru*f*-(2→1)]_13_-*α*-D-Glc*p* | C_84_H_142_O_71_ | 2286.75 | *C. heracleifolia* | Rhizome | [75] |
| 328 | *β*-D-Fru*f*-(2→1)-*β*-D-Fru*f* | C_12_H_22_O_11_ | 342.30 | *C. heracleifolia* | Rhizome | [75] |
| 329 | [*β*-D-Fru*f*-(2→1)]_2_-*β*-D-Fru*f* | C_18_H_32_O_16_ | 504.17 | *C. heracleifolia* | Rhizome | [75] |
| 330 | [*β*-D-Fru*f*-(2→1)]_3_-*β*-D-Fru*f* | C_24_H_42_O_21_ | 666.22 | *C. heracleifolia* | Rhizome | [75] |
| 331 | [*β*-D-Fru*f*-(2→1)]_4_-*β*-D-Fru*f* | C_30_H_52_O_26_ | 828.28 | *C. heracleifolia* | Rhizome | [75] |
| 332 | [*β*-D-Fru*f*-(2→1)]_5_-*β*-D-Fru*f* | C_36_H_62_O_31_ | 990.33 | *C. heracleifolia* | Rhizome | [75] |
| 333 | [*β*-D-Fru*f*-(2→1)]_6_-*β*-D-Fru*f* | C_42_H_72_O_36_ | 1152.38 | *C. heracleifolia* | Rhizome | [75] |
| 334 | [*β*-D-Fru*f*-(2→1)]_7_-*β*-D-Fru*f* | C_48_H_82_O_41_ | 1314.43 | *C. heracleifolia* | Rhizome | [75] |
| 335 | [*β*-D-Fru*f*-(2→1)]_8_-*β*-D-Fru*f* | C_54_H_92_O_46_ | 1476.49 | *C. heracleifolia* | Rhizome | [75] |
| 336 | [*β*-D-Fru*f*-(2→1)]_9_-*β*-D-Fru*f* | C_60_H_102_O_51_ | 1638.54 | *C. heracleifolia* | Rhizome | [75] |

Continued

| Number | Chemical constituents | Molecular formula | MW | Source plants | Part of the plants | References |
| --- | --- | --- | --- | --- | --- | --- |
| 337 | [*β*-D-Fru*f*-(2→1)]_10_-*β*-D-Fru*f* | C_66_H_112_O_56_ | 1800.59 | *C. heracleifolia* | Rhizome | [75] |
| 338 | [*β*-D-Fru*f*-(2→1)]_11_-*β*-D-Fru*f* | C_72_H_122_O_61_ | 1962.65 | *C. heracleifolia* | Rhizome | [75] |
| 339 | [*β*-D-Fru*f*-(2→1)]_12_-*β*-D-Fru*f* | C_78_H_132_O_66_ | 2124.70 | *C. heracleifolia* | Rhizome | [75] |
| 340 | [*β*-D-Fru*f*-(2→1)]_13_-*β*-D-Fru*f* | C_84_H_142_O_71_ | 2286.75 | *C. heracleifolia* | Rhizome | [75] |
| 341 | cimitriteromone H | C59H86O21 | 1130.57 | *C.* foetida | Rhizome | [76] |
| 342 | cimitriteromone F | C57H82O19 | 1070.55 | *C.* foetida | Rhizome | [76] |
| 343 | cimitriteromone I | C59H84O20 | 1112.56 | *C.* foetida | Rhizome | [76] |
| 344 | cimitriteromone A | C59H84O20 | 1112.56 | *C.* foetida | Rhizome | [76] |
| 345 | cimitriteromone B | C59H84O20 | 1112.56 | *C.* foetida | Rhizome | [76] |
| 346 | cimitriteromone C | C53H74O18 | 998.49 | *C.* foetida | Rhizome | [76] |
| 347 | cimitriteromone D | C59H86O21 | 1130.57 | *C.* foetida | Rhizome | [76] |
| 348 | cimitriteromone E | C59H84O21 | 1128.55 | *C.* foetida | Rhizome | [76] |

Table S4. The pharmacological activities, extract, dose, model and results of Cimicifugae Rhizoma are summarized.

| Activity | Extract/Compound | Dose/IC_50_ | Animal/Cell lines | Model/Diseases | Results | Reference |
| --- | --- | --- | --- | --- | --- | --- |
| anti-inflammatory | ferulic acid | 20, 40, 80 mg/kg | male ICR mice | chronic depressive-like model | antidepressant effect of ferulic acid was related to anti-inflammatory effect | [77] |
| anti-inflammatory | 24-*epi*-24-*O*-acetyl-7,8-didehydro-shengmanol,  25-*O*-acetyl-7,8-didehydrocimigenol | IC_50_ = 1.91±0.13, 1.27±0.05 μM | bone marrow-derived dendritic cells (BMDCs) | BMDCs stimulated by lipopolysaccharide (LPS) | they had good anti-inflammatory effect by inhibiting the expression of IL-12p40, IL-6, and TNF-*α* | [78] |
| anti-inflammatory | cimicifugic acid D  cimicifugic acid A | IC_50_ = 70.15, 27.42 μM， respectively | RAW264.7 cells | RAW264.7 cells treated by LPS | cimicifugic acid D had good anti-inflammatory by inhibiting PGE_2_ production | [69] |
| anti-inflammatory | 23-*O*-acetylshengmanol-3-*O*-*α*-L-arabinoside | 5, 25 mg/kg | C57BL/6 male mice,  RAW264.7 cell | LPS-stimulated acute lung injury model | playing anti-inflammatory effect through downregulating the NLRP3/caspase-1, I*κ*B/NF-*κ*B and MAPKs/AP-1 pathway | [79] |
| anti-inflammatory | 60% ethanol of CR extract | 30, 100 mg/kg for 6 days | Female BALB/c mice | asthmatic induced by OVA | it can effectively inhibit OVA-induced airway inflammation and oxidative stress | [80] |
| anti-atherosclerosis | yunnanterpene G | 0, 10, 25, 50 µM | Human monocytic THP-1 cells | atherosclerosis | inhibiting the expression of CD147 and MMPs to stabilize atherosclerotic plaque | [59] |

Continued

| Activity | Extract/Compound | Dose/IC_50_ | Animal/Cell lines | Model/Diseases | Results | Reference |
| --- | --- | --- | --- | --- | --- | --- |
| anti-depressant | XMT | 20, 40, 80 mg/kg/day in mice  10, 20, 40 mg/kg/day in rats | female Kunming (KM) mice, SD rats | forced swimming test,tail suspension test, 5-HTP-induced head-twitch test | XMT had antidepressant effect | [81] |
| antioxidant | ferulic acid | 10, 50 mg/kg | wistar albino rats | ormaldehyde-induced hepatotoxicity | FA inhibited the production of chondrex and improved the activity of antioxidant enzymes to protect the liver | [82] |
| antioxidant | 2-feruloyl piscidic acid | IC_50_ = 9.33±1.67 µM | DPPH | free radical scavenging | phenolic compounds in *Cimicifuga* could scavenge free radicals | [63] |
| anti-tumor | ethyl acetate layer of CR | 10, 20, 40 μg/mL  15, 30, 60 μg/mL, respectively | MCF-7, MDA-MB-231 cells | breast cancer | significantly inhibit the proliferation, migra-  tion and invasion of cells | [83] |
| anti-tumor | actein | 10, 15 μM, respectively,  15 mg/kg, once every two days for 21 days | human gastric cancer cell lines, SNU-216 and AGS, and human gastric epithelial cell GES1,  athymic nude mice | gastric cancer,  athymic nude model | repressing Bcl-2, Mcl-1 and increasing Bad, Bak expression to inhibit the growth of gastric cancer and promote apoptosis | [84] |

Continued

| Activity | Extract/Compound | Dose/IC_50_ | Animal/Cell lines | Model/Diseases | Results | Reference |
| --- | --- | --- | --- | --- | --- | --- |
| anti-tumor | cimigenoside | 5, 10, 20 μM | MCF-7, MCF-10A, T47D，MDA-MB-231, HEK293 cells | breast cancer | cimigenoside inhibited the proliferation of breast cancer by *γ*-secretase/Notch axis | [85] |
| anti-tumor | actein | 0, 1, 2.5, 5, 10, 15, 20, 25, 30 μM | A549, H1975, BEAS2B, AML12， BRL3A cells | non-small-cell lung cancer NSCLC | activating caspase 3-signaling pathway and suppressing NSCLC growth in a p53-dependent manner | [86] |
| anti-tumor | actein | 5, 10, 20 μg/mL | CAL-27 and SCC-9 human OSCC cell lines | oral squamous cell carcinoma | actein inhibited cancer cell proliferation through Akt/FoxO1 pathway | [87] |
| anti-tumor | isoferulic acid | 5, 15, 45 µM | Jurkat，K562，raji cells | hematologic malignancy | through G2/M‑phase arrest and inhibition of Akt/mTOR signaling to repress cells growth | [88] |
| anti-tumor | cimigenoside | 0, 1, 2, 5 µmol/L | A549 cells | lung cancer | repressing cell proliferation， inducing apoptosis through NF-*κ*B pathway | [89] |
| anti-tumor | 25-*O*-acetyl-7,8-didehydrocimi  genol 3-*O*-*β*-D-xylopyranoside | 20, 24, 28 µM | HepG2/ADM,  HepG2 cells | hepatocellular carcinoma | inhibiting autophagic degradation by overcoming multidrug resistance through Akt-mediated | [90] |

Continued

| Activity | Extract/Compound | Dose/IC_50_ | Animal/Cell lines | Model/Diseases | Results | Reference |
| --- | --- | --- | --- | --- | --- | --- |
| anti-tumor | cimigenol | 0, 1, 5, 10 µM | HT-29 cells | colorectal cancer | cimigenol increased the expression of cleaved-caspase-8 and -3, the cleavage of ADP-ribose PARP to promote apoptosis | [91] |
| antiviral | fukinolic acid  cimicifugic acid A | EC_50_ = 4.3±0.1, 21.3±11 μg /mL, respectively | Human enterovirus 71 (EV-A71) | enterovirus | they had significant anti-EV-A71 activity | [92] |
| antiviral | water extract of CR | IC_50_ = 67.3, 31.0 μg/mL, respectively | HEp-2, A549 Cell Lines | human respiratory syncytial virus HRSV  induced plaque formation | inhibiting viral attachment and HRSV internalization and  stimulating to secrete IFN-*β* to counteract viral infection | [93] |
| antiviral | cimicifugin | IC_50_ = 5.4, 38.6 μg/mL, respectively | A549, HEp-2 cells | culture human respiratory syncytial virus | stimulating epithelial cells to secrete IFN-*β* to counteract viral infection | [94] |
| ease pain | XMT | 1talet everyday for 6 months | 96 early postmenopausal women in clinical | breast pain | the incidence and duration of breast pain were lower than upon treatment with E2 plus cyclic MPA or m-P | [95] |
| hypolipidemic | 23-*epi*-26-deoxyactein | 0.1, 1, 5, 10, 20, 50 μM  1, 5 mg/kg/d for 12 weeks | 3T3-L1 preadipocytes, C57BL/6 mice | 3T3-L1 preadipocytes cultured in high glucose, fed on high-fat diet | inhibiting 3T3-L1 forming grease and activating AMPK and SIRT1-FOXO1 pathway to promote adipocyte lipolysis | [96] |

Continued

| Activity | Extract/Compound | Dose/IC_50_ | Animal/Cell lines | Model/Diseases | Results | Reference |
| --- | --- | --- | --- | --- | --- | --- |
| hypolipidemic | cimicifugoside H-2;  cimicifugoside H-3 | 10 μM | 3T3-L1 cells | 3T3-L1 preadipocytes cultured in high glucose | repressing fat accumulation in 3T3-L1 | [13] |
| increasing the osteogenic differentiation | water extract of CR | 0.1, 1, 10 μg/mL | human stem cells derived from gingiva | effects on the osteogenic and adipogenic differentiation of stem cells | Promoting the proliferation of stem cells derived from the gingiva | [97] |
| neuroprotective | cimiciphenone, *E* -3- 3'-methyl-2'-butenylidene -1-methyl-2-indolinone；  *E* -3- 3'-methyl-2'-butenylidene -2-indolinone | IC_50_ = 16.7±1.9, 13.8±1.5, 6.5±2.5 µM, respectively | acetylcholinesterase  butyrylcholinesterase | alzheimer’s disease | they all showed strong neuroprotective activity | [98] |
| neuroprotective | 24-*epi*-24-*O*-acetyl-7,8-didehydroshengmanol 3-*O*-*β*-D-xylopyranoside | 0, 2.5, 5, 10 µM,  1.25, 2.5, 5 mg/kg/day for 7 days | APP-CHO cells,  Male ICR mice | alzheimer’s disease | improving behavioral defects and inhibiting AChE activity | [99] |

Continued

| Activity | Extract/Compound | Dose/IC_50_ | Animal/Cell lines | Model/Diseases | Results | Reference |
| --- | --- | --- | --- | --- | --- | --- |
| neuroprotective | 4'-methoxyl-3'-hydroxy-carboxybenzoyl isoferulic acid anhydride | 1, 5, 10 µM | Pheochromocytoma PC12 cells | oxidative stress induced PC12 cell damage model | polyphenolic compounds displayed marked neuro-protective effect | [100] |
| relieving menopausal symptoms | 60% ethanol Cimicifugae Rhizoma extract | 60, 180 mg/kg last 12 weeks | female SD rats | ovariectomized female rats | improving the OVX-induced metabolic disorders and alleviating menopause symptoms. | [101] |
| treating skin diseases | Water extraction of CR and *Smilax glabra* | 3.85, 7.7, 15.4 g/kg/day | Balb/c mice | imiquimod-induced psoriasis-like mouse model | inhibiting the expression of CCL2, CCL7, IL1F6, IL-17 and other factors to alleviate psoriasis-like dermatitis | [102] |

Abb. Cimicifugae Rhizoma CR; bone marrow-derived dendritic cells BMDCs; lipopolysaccharide LPS; Interleukin-12p40 IL-12p40; Interleukin-6 IL-6; Tumour Necrosis Factor alpha TNF-α; NOD-like receptor thermal protein domain associated protein 3 NLRP3; NF-kappa B NF-κB; non-small-cell lung cancer NSCLC; Prostaglandin E_2_  PEG_2_; medroxyprogesterone MPA; sirtuin1 SIRT1; Protein kinase B Akt; micronized progesterone m-P; forkhead transcription factor O1 FOXO1; AMP-activated protein kinase AMPK; Interferon-β IFN-β; mammalian target of rapamycin mTOR; poly ADP-ribose polymerase PARP.

**Reference**

1. iPlant. China. http://www.iplant.cn/. Accessed 16 June 2023.

2. Zhang Z, Liu Q, Xi R, Xu H. Application of Mahuang Shengma Decoction in the Treatment of Chronic Heart Failure Complicated with Pulmonary Infection Using the Method of Correspondence of Decoction and Pathogenesis. Journal of Emergency in Traditional Chinese Medicine. 2022; 31: (05), 812-814+823. https://doi.org/10.3969/j.issn.1004-745X.2022.05.015.

3. Zhongyishijia. https://www.zysj.com.cn/. Accessed 12 Jul 2023.

4. Zhongyibaodian. http://zhongyibaodian.com/. Accessed 23 Jun 2023.

5. Commission SP. Pharmacopoeia of the People's Republic of China (The First Division). 2020; 37: (02), 126-129. https://doi.org/10.13375/j.cnki.wcjps.2022.02.004.

6. Li T, Meng Q, Hua E, Sun Y. Study on the cycloartane triterpenoids from Cimicifuga dahurica and their biological activity. Chinese Journal of Medicinal Chemistry. 2021; 31: (07), 520-531. https://doi.org/10.14142/j.cnki.cn21-1313/r.2021.07.006.

7. Li JX, Kadota S, Hattori M, Yoshimachi S, Shiro M, Oogami N, Mizuno H, Namba T. CONSTITUENTS OF CIMICIFUGAE RHIZOMA .1. ISOLATION AND CHARACTERIZATION OF 10 NEW CYCLOARTENOL TRITERPENES FROM CIMICIFUGA-HERACLEIFOLIA KOMAROV. Chem Pharm Bull. 1993; 41: (5), 832-841.

8. Pang QQ, Li T, Liu LX, Shi DF, Yao XS, Li HB, Yu Y. Systematically identifying the anti-inflammatory constituents of Cimicifuga dahurica by UPLC-Q/TOF-MS combined with network pharmacology analysis. Biomed Chromatogr. 2021; 35: (12). https://doi.org/10.1002/bmc.5177.

9. Cao P, Pu XF, Peng SL, Zhang XR, Ding LS. Chemical constituents from Cimicifuga foetida. Journal of Asian Natural Products Research. 2005; 7: (2), 145-149. https://doi.org/10.1080/1028602042000204081.

10. Thao NP, Luyen BTT, Lee JS, Kim JH, Dat NT, Kim YH. Inhibition Potential of Cycloartane-Type Glycosides from the Roots of Cimicifuga dahurica against Soluble Epoxide Hydrolase. J Nat Prod. 2017; 80: (6), 1867-1875. https://doi.org/10.1021/acs.jnatprod.7b00166.

11. Yoshimitsu H, Nishida M, Sakaguchi M, Nohara T. Two new 15-deoxycimigenol-type and three new 24-epi-cimigenol-type glycosides from Cimicifuga Rhizome. Chem Pharm Bull. 2006; 54: (9), 1322-1325. https://doi.org/10.1248/cpb.54.1322.

12. Pang QQ, Mei YD, Zhang YC, Liu LX, Shi DF, Pan DB, Yao XS, Li HB, Yu Y. Three new cycloart-7-ene triterpenoid glycosides from Cimicifuga dahurica and their anti-inflammatory effects. Natural Product Research. 2021; 35: (21), 3634-3643. https://doi.org/10.1080/14786419.2020.1719487.

13. Shi QQ, Lu SY, Li DS, Lu J, Zhou L, Qiu MH. Cycloartane triterpene glycosides from rhizomes of Cimicifuga foetida L. with lipid-lowering activity on 3T3-L1 adipocytes. Fitoterapia. 2020; 145. https://doi.org/10.1016/j.fitote.2020.104635.

14. Wang HY, Nian Y, Ma CY, Liu JQ, Song YH, Zhou L, Qiu MH. Four New 9,19-Cyclolanostane Triterpenes from the Rhizomes of Cimicifuga foetida Collected in Yulong. Chinese J Chem. 2012; 30: (6), 1265-1268. https://doi.org/10.1002/cjoc.201200193.

15. Guo YQ, Yin T, Wang XM, Zhang F, Pan GX, Lv H, Wang XR, Orgah JO, Zhu Y, Wu HH. Traditional uses, phytochemistry, pharmacology and toxicology of the genus Cimicifuga: A review. J Ethnopharmacol. 2017; 209, 264-282. https://doi.org/10.1016/j.jep.2017.07.040.

16. Lu L, Chen JC, Li Y, Qing C, Wang YY, Nian Y, Qiu MH. Studies on the constituents of Cimicifuga foetida collected in Guizhou Province and their cytotoxic activities. Chem Pharm Bull (Tokyo). 2012; 60: (5), 571-7. https://doi.org/10.1248/cpb.60.571.

17. Wang XY, Li CJ, Ma J, Li C, Chen FY, Wang N, Shen CJ, Zhang DM. Cytotoxic 9,19-cycloartane type triterpenoid glycosides from the roots of Actaea dahurica. Phytochemistry. 2019; 160, 48-55. https://doi.org/10.1016/j.phytochem.2019.01.004.

18. Thao NP, Kim JH, Thuy Luyen BT, Dat NT, Kim YH. In silico investigation of cycloartane triterpene derivatives from Cimicifuga dahurica (Turcz.) Maxim. roots for the development of potent soluble epoxide hydrolase inhibitors. Int J Biol Macromol. 2017; 98, 526-534. https://doi.org/10.1016/j.ijbiomac.2017.02.023.

19. Zhang QW, Ye WC, Che CT, Zhao SX. A new cycloartane saponin from Cimicifuga acerina. Journal of Asian Natural Products Research. 1999; 2: (1), 45-49. https://doi.org/10.1080/10286029908039890.

20. Song Y, Nian Y, Ma W, Qiu M. Studies on Chemical Constituents of the Rhizomes of Cimicifuga dahurica. Journal of Yunnan University of Traditional Chinese Medicine. 2013; 36: (03), 31-35. https://doi.org/10.19288/j.cnki.issn.1000-2723.2013.03.009.

21. Nian Y, Zhang YL, Chen JC, Lu L, Qiu MH, Qing C. Cytotoxic Chemical Constituents from the Roots of Cimicifuga fetida. J Nat Prod. 2010; 73: (2), 93-98. https://doi.org/10.1021/np9003855.

22. Hu LF, Song XJ, Nagai T, Yamamoto M, Dai Y, He LL, Kiyohara H, Yao XS, Yao ZH. Chemical profile of Cimicifuga heracleifolia Kom. And immunomodulatory effect of its representative bioavailable component, cimigenoside on Poly(I: C)-induced airway inflammation. J Ethnopharmacol. 2021; 267. https://doi.org/10.1016/j.jep.2020.113615.

23. Pan R, Chen D, Si J, Zhao X, Shen L. Studies on the triterpenoid constituents from the aerial part of Cimicifuga foetida L. Acta Pharmaceutica Sinica. 2002: (02), 117-120. https://doi.org/10.16438/j.0513-4870.2002.02.009.

24. Gao JC, Huang F, Zhang JC, Zhu GY, Yang MS, Xiao PG. Cytotoxic cycloartane triterpene saponins from Actaea asiatica. J Nat Prod. 2006; 69: (10), 1500-1502. https://doi.org/10.1021/np060113h.

25. Sun LR, Yan J, Nian Y, Zhou L, Zhang HJ, Qiu MH. New triterpene diglycosides from the rhizome of Cimifuga foetida. Molecules. 2008; 13: (8), 1712-1721. https://doi.org/10.3390/molecules13081712.

26. Sun LR, Qing C, Zhang YL, Jia SY, Li ZR, Pei SJ, Qiu MH, Gross ML, Qiu SX. Cimicifoetisides A and B, two cytotoxic cycloartane triterpenoid glycosides from the rhizomes of Cimicifuga foetida, inhibit proliferation of cancer cells. Beilstein Journal of Organic Chemistry. 2007; 3. https://doi.org/10.1186/1860-5397-3-3.

27. Zhu GL, Nian Y, Zhu DF, Wan LS, Bao NM, Wang WH, Zhou L, Qiu MH. Cytotoxic 9,19-cycloartane triterpenoids from the roots of Cimicifuga foetida L. Phytochemistry Letters. 2016; 18, 105-112. https://doi.org/10.1016/j.phytol.2016.06.002.

28. Pan RL, Chen DH, Si JY, Zhao XH, Li Z, Cao L. Immunosuppressive effects of new cyclolanostane triterpene diglycosides from the aerial part of Cimicifuga foetida. Arch Pharm Res. 2009; 32: (2), 185-90. https://doi.org/10.1007/s12272-009-1133-1.

29. Shi Q, Lu S, Li D, Lu J, Zhou L, Qiu M. Cycloartane triterpene glycosides from rhizomes of Cimicifuga foetida L. with lipid-lowering activity on 3T3-L1 adipocytes. Fitoterapia. 2020; 145, 104635. https://doi.org/10.1016/j.fitote.2020.104635.

30. Pan R, Chen D, Si J, Zhao X, Shen L. Studies on the New Triterpenoid Saponin of the Aerial Part of Cimifuga foetida. China Journal of Chinese Materia Medica. 2003: (03), 41-43.

31. Ali Z, Khan SI, Khan IA. Phytochemical study of actaea rubra and biological screenings of isolates. Planta Med. 2006; 72: (14), 1350-2. https://doi.org/10.1055/s-2006-951696.

32. Nian Y, Zhang XM, Li Y, Wang YY, Chen JC, Lu L, Zhou L, Qiu MH. Cycloartane triterpenoids from the aerial parts of Cimicifuga foetida Linnaeus. Phytochemistry. 2011; 72: (11-12), 1473-1481. https://doi.org/10.1016/j.phytochem.2011.03.022.

33. Li JX, Li JF, Chen SJ, Yu ZY. An unusual metabolite of cimicidol-3-O-beta-D-xyloside from Cimicifugae rhizoma by rat intestinal bacteria. Chem Biodivers. 2008; 5: (2), 290-298. https://doi.org/DOI 10.1002/cbdv.200890026.

34. Huyen CTT, Luyen BTT, Khan GJ, Oanh HV, Hung TM, Li HJ, Li P. Chemical Constituents from Cimicifuga dahurica and Their Anti-Proliferative Effects on MCF-7 Breast Cancer Cells. Molecules. 2018; 23: (5). https://doi.org/10.3390/molecules23051083.

35. Li JX, Kadota S, Hattori M, Yoshimachi S, Shiro M, Oogami N, Mizuno H, Namba T. Constituents of Cimicifugae Rhizoma Isolation and characterization of 10 new cycloartenol triterpenes from cimicifuga-heracleifolia komarov. Chem Pharm Bull. 1993; 41: (5), 832-841.

36. Liu YR, Wu ZJ, Li CT, Xi FM, Sun LN, Chen WS. Heracleifolinosides A-F, new triterpene glycosides from Cimicifuga heracleifolia, and their inhibitory activities against hypoxia and reoxygenation. Planta Medica. 2013; 79: (3-4), 301-307. https://doi.org/10.1055/s-0032-1328174.

37. Li CJ, Chen DH, Xiao PG. Chemical constituents of traditional Chinese drug "sheng-ma" (Cimicifuga dahurica). Yao Xue Xue Bao. 1993; 28: (10), 777-81.

38. Sakurai N, Koeda M, Inoue T, Nagai M. Studies on the Chinese crude drug "shoma". 8.2 New triterpenol bisdesmosides, 3-arabinosyl-24-O-acetylhydroshengmanol 15-glucoside and 3-xyloside-24-O-acetylhydroshengmanol 15-glucoside, from cimicifuga-dahurica. Chem Pharm Bull. 1994; 42: (1), 48-51.

39. Nian Y, Chen JC, Lu L, Zhang XM, Zhou L, Qiu MH. Four New 9,19-Cyclolanostane Derivatives from the Rhizomes of Cimicifuga yunnanensis HSIAO. Helvetica Chimica Acta. 2009; 92: (1), 112-120. https://doi.org/10.1002/hlca.200800231.

40. Kuang HX, Su Y, Wang QH, Wu L, Yang BY, Wang ZB, Xia YG. Three New Cycloartenol Glycosides from the Roots of Cimicifuga simplex. Planta Medica. 2012; 78: (6), 622-NUL_7. https://doi.org/10.1055/s-0031-1298224.

41. Kusano A, Shibano M, Kusano G, Miyase T. Studies on the constituents of Cimicifuga species .19. eight new glycosides from Cimicifuga simplex WORMSK. Chem Pharm Bull. 1996; 44: (11), 2078-2085.

42. Wang XY, Li CJ, Ma J, Li C, Huang JW, Wang N, Shen CJ, Zhang DM. Cytotoxic 9,19-cycloartane Triterpenoids from the Roots of Actaea dahurica. Fitoterapia. 2019; 137, 104262. https://doi.org/10.1016/j.fitote.2019.104262.

43. Sakurai N, Koeda M, Aoki Y, Nagai M. Studies on the Chinese crude drug "shoma".10.3 New trinor-9,19-cyclolanostanol xylosides, cimicifugosides H-3, H-4, and H-6, from cimicifuga rhizome and transformation of cimicifugoside H-1 into cimifugosides H-2, H-3 and H-4. . Chem Pharm Bull. 1995; 43: (9), 1475-1482.

44. Zhu DF, Nian Y, Wang HY, Zhang ZR, Song YB, Li RT, Qiu MH. New 9, 19-cycloartane triterpenoid from the root of Cimicifuga foetida. Chinese Journal of Natural Medicines. 2014; 12: (4), 294-296. https://doi.org/10.1016/s1875-5364(14)60057-1.

45. Chen JY, Li PL, Tang XL, Wang SJ, Jiang YT, Shen L, Xu BM, Shao YL, Li GQ. Cycloartane triterpenoids and their glycosides from the rhizomes of Cimicifuga foetida. J Nat Prod. 2014; 77: (9), 1997-2005. https://doi.org/10.1021/np500249v.

46. Lu L, Chen JC, Song HJ, Li Y, Nian Y, Qui MH. Five New Triterpene Bisglycosides with Acyclic Side Chains from the Rhizomes of Cimicifuga foetida L. Chem Pharm Bull. 2010; 58: (5), 729-733. https://doi.org/10.1248/cpb.58.729.

47. Kusano A, Shibano M, Kusano G. Studies on the constituents of Cimicifuga species. XXVII. Malonyl cyclolanostanol glycosides from the underground parts of Cimicifuga simplex Wormsk. Chem Pharm Bull. 1999; 47: (8), 1175-1179.

48. Lu NH, Yang YR, Li XF, Liu HL, Zhao ZR, Du YR. New cycloartane triterpenes from the roots of Cimicifuga foetida. Phytochemistry Letters. 2021; 42, 109-116. https://doi.org/10.1016/j.phytol.2021.01.009.

49. Nian Y, Wang HY, Su J, Zhou L, Qiu MH. A cytotoxic 4α-methyl steroid from the aerial parts of Cimicifuga foetida L. Fitoterapia. 2012; 83: (2), 293-7. https://doi.org/10.1016/j.fitote.2011.11.001.

50. Sun LR, Yan J, Zhou L, Li ZR, Qiu MH. Two New Triterpene Glycosides with Monomethyl Malonate Groups from the Rhizome of Cimifuga foetida L. Molecules. 2011; 16: (7), 5701-5708. https://doi.org/10.3390/molecules16075701.

51. Kadota S, Li JX, Tanaka K, Namba T. Constituents of Cimicifugae Rhizoma .2. Isolation and Structures of New Cycloartenol Triterpenoids and Related-Compounds from Cimicifuga-Foetida L. Tetrahedron. 1995; 51: (4), 1143-1166. https://doi.org/Doi 10.1016/0040-4020(94)01015-R.

52. Nishida M, Yoshimitsu H. Six New Cycloartane Glycosides from Cimicifuga Rhizome. Chem Pharm Bull. 2011; 59: (10), 1243-1249.

53. Liu Y, Chen DH, Si JY, Tu GZ, An DG. Two new cyclolanostanol xylosides from the aerial parts of Cimicifuga dahurica. J Nat Prod. 2002; 65: (10), 1486-1488. https://doi.org/10.1021/np020130g.

54. Nian Y, Zhu H, Tang WR, Luo Y, Du J, Qiu MH. Triterpenes from the aerial parts of Cimicifuga yunnanensis and their antiproliferative effects on p53(N236S) mouse embryonic fibroblasts. J Nat Prod. 2013; 76: (5), 896-902. https://doi.org/10.1021/np4000262.

55. Zhu DF, Zhu GL, Kong LM, Bao NM, Zhou L, Nian Y, Qiu MH. Cycloartane Glycosides from the Roots of Cimicifuga foetida with Wnt Signaling Pathway Inhibitory Activity. Natural products and bioprospecting. 2015; 5: (2), 61-67. https://doi.org/10.1007/s13659-015-0053-7.

56. Chen SN, Fabricant DS, Lu ZZ, Fong HH, Farnsworth NR. Cimiracemosides I-P, new 9,19-cyclolanostane triterpene glycosides from Cimicifuga racemosa. J Nat Prod. 2002; 65: (10), 1391-7. https://doi.org/10.1021/np0200818.

57. Zhou W, Lu Q, Lv C, Zhang X, Qin R, Lu J. Isolation and identification of the chemical constitu ents from the rhizomes of Cimicifuga dahurica (Turcz.) Maxim．. Journal of Shenyang Pharmaceutical University. 2018; 35: (04), 269-273. https://doi.org/10.14066/j.cnki.cn21-1349/r.2018.04.004.

58. Yoshimitsu H, Nishida M, Nohara T. Three new 15,16-seco-cycloartane glycosides from Cimicifuga Rhizome. Chem Pharm Bull. 2007; 55: (5), 789-792. https://doi.org/10.1248/cpb.55.789.

59. Lu NH, Zhang ZW, Guo RW, Yang LX, Song YX, Ye JS, Shi YK. Yunnanterpene G, a spiro-triterpene from the roots of Cimicifuga foetida, downregulates the expression of CD147 and MMPs in PMA differentiated THP-1 cells. RSC Adv. 2018; 8: (27), 15036-15043. https://doi.org/10.1039/c8ra01895b.

60. Zhao X, Chen D, Si J, Pan R, Shen L. Studies on the phenolic acid constituents from Chinese medicine "shengma", Rhizome of Cimicifuga foetida L. Acta Pharmaceutica Sinica. 2002: (07), 535-538. https://doi.org/10.16438/j.0513-4870.2002.07.008.

61. Fan MX, Qin KM, Ding F, Huang YT, Wang XL, Cai BC. Identification and differentiation of major components in three different "Sheng-ma" crude drug species by UPLC/Q-TOF-MS. Acta Pharm Sin B. 2017; 7: (2), 185-192. https://doi.org/10.1016/j.apsb.2016.11.002.

62. Lu Q, Li HB, Pang QQ, Zhang WY, Su ZZ, Pan DB, Yao XS, Yu Y. New phenylpropanoid allopyranosides from the rhizomes of Cimicifuga dahurica. Bioorg Med Chem Lett. 2019; 29: (14), 1774-1778. https://doi.org/10.1016/j.bmcl.2019.05.011.

63. Qin RL, Zhao Y, Zhao YD, Zhou WR, Lv CN, Lu JC. Polyphenolic compounds with antioxidant potential and neuro-protective effect from Cimicifuga dahurica (Turcz.) Maxim. Fitoterapia. 2016; 115, 52-56. https://doi.org/10.1016/j.fitote.2016.09.016.

64. Qin R, Lv C, Yu Y, Lu J. Isolation and Identification of Chemical Constituents of Phenolic Acid from Cimicifuga dahurica. Chinese Journal of Experimental Traditional Medical Formulae. 2019; 25: (21), 112-117. https://doi.org/10.13422/j.cnki.syfjx.20191917.

65. Thao NP, Luyen BTT, Lee JS, Kim JH, Kim YH. Soluble epoxide hydrolase inhibitors of indolinone alkaloids and phenolic derivatives from Cimicifuga dahurica (Turcz.) Maxim. Bioorg Med Chem Lett. 2017; 27: (8), 1874-1879. https://doi.org/10.1016/j.bmcl.2017.02.013.

66. Lu Q, Li H, Yao X, Yu Y. Chemical constituents from rhizomes of Cimicifuga dahurica. Chinese Herbal Medicine. 2019; 50: (14), 3261-3268. https://doi.org/10.7501/j.issn.0253-2670.2019.14.001

67. Yim SH, Kim HJ, Jeong N, Park KD, Lee YJ, Cho SD, Lee IS. Structure-Guided Identification of Novel Phenolic and Phenolic Amide Allosides from the Rhizomes of Cimicifuga heracleifolia. B Korean Chem Soc. 2012; 33: (4), 1253-1258. https://doi.org/10.5012/bkcs.2012.33.4.1253.

68. Thao NP, Luyen BT, Lee JS, Kim JH, Kim YH. Soluble epoxide hydrolase inhibitors of indolinone alkaloids and phenolic derivatives from Cimicifuga dahurica (Turcz.) Maxim. Bioorg Med Chem Lett. 2017; 27: (8), 1874-1879. https://doi.org/10.1016/j.bmcl.2017.02.013.

69. Lu Q, Zhang WY, Pan DB, Shi DF, Pang QQ, Li HB, Yao XJ, Yao ZH, Yu Y, Yao XS. Phenolic acids and their glycosides from the rhizomes of Cimicifuga dahurica. Fitoterapia. 2019; 134, 485-492. https://doi.org/10.1016/j.fitote.2019.03.023.

70. Nuntanakorn P, Jiang B, Einbond LS, Yang H, Kronenberg F, Weinstein IB, Kennelly EJ. Polyphenolic constituents of Actaea racemosa. J Nat Prod. 2006; 69: (3), 314-318. https://doi.org/10.1021/np0501031.

71. Wang Z, Wang Q, Zhang M, Hu X, Ding G, Jiang M, Bai G. Cimicifugamide from Cimicifuga rhizomes functions as a nonselective β-AR agonist for cardiac and sudorific effects. Biomed Pharmacother. 2017; 90, 122-130. https://doi.org/10.1016/j.biopha.2017.03.058.

72. Hao Y, Luo W, Jiang G, Lv C, Lu J. Isolation and identification of the chemical constituents from n-BuOH extracts of the aerial parts of Cimicifuga dahurica (Turcz.) Maxim．. Journal of Shenyang University. 2019; 36: (06), 482-486+491. https://doi.org/10.14066/j.cnki.cn21-1349/r.2019.06.003.

73. Zhou C, Yu Y, Sheng R, Mo JX, Huang M, Ouyang L, Gan LS. Cimicifoetones A and B, Dimeric Prenylindole Alkaloids as Black Pigments of Cimicifuga foetida. Chemistry, an Asian journal. 2017; 12: (12), 1277-1281. https://doi.org/10.1002/asia.201700348.

74. Ma LJ, Wang YH, Tang GH, Wang YL, Ma C, Dastmalchi K, Kennelly EJ, Long CL. New Monoterpene Lactones from Actaea cimicifuga. Planta Medica. 2013; 79: (3-4), 308-311. https://doi.org/10.1055/s-0032-1328127.

75. Cui L, Wu J, Wang X, Yang X, Ye Z, Mayo KH, Sun L, Zhou Y. Purification and identification of oligosaccharides from Cimicifuga heracleifolia Kom. rhizomes. Food chemistry: X. 2023; 18, 100706. https://doi.org/10.1016/j.fochx.2023.100706.

76. Shi QQ, Gao Y, Lu J, Zhou L, Qiu MH. Two new triterpenoid-chromone hybrids from the rhizomes of Actaea cimicifuga L. (syn. Cimicifuga foetida L.) and their cytotoxic activities. Nat Prod Res. 2022; 36: (1), 193-199. https://doi.org/10.1080/14786419.2020.1775228.

77. Liu YM, Shen JD, Xu LP, Li HB, Li YC, Yi LT. Ferulic acid inhibits neuro-inflammation in mice exposed to chronic unpredictable mild stress. International Immunopharmacology. 2017; 45, 128-134. https://doi.org/10.1016/j.intimp.2017.02.007

78. Thao NP, Lee YS, Luyen B, Oanh HV, Kim YH. Chemicals from Cimicifuga dahurica and Their Inhibitory Effects on Pro-inflammatory Cytokine Production by LPS-stimulated Bone Marrow-derived Dendritic Cells. Natural Product Sciences. 2018; 24: (3), 194. https://doi.org/10.20307/nps.2018.24.3.194.

79. Chen CY, Li L, Liu XM, Zhang DQ, Liu Y, Li YH. 23-O-acetylshengmanol-3-O-alpha-L-arabinoside alleviates lipopolysaccharide-induced acute lung injury through inhibiting I kappa B/NF-kappa B and MAPK/AP-1 signaling pathways. J Ethnopharmacol. 2023; 300. https://doi.org/10.1016/j.jep.2022.115725.

80. Lim JO, Song KH, Lee IS, Lee SJ, Kim WI, Pak SW, Shin IS, Kim T. Cimicifugae Rhizoma Extract Attenuates Oxidative Stress and Airway Inflammation via the Upregulation of Nrf2/HO-1/NQO1 and Downregulation of NF-kappa B Phosphorylation in Ovalbumin-Induced Asthma. Antioxidants-Basel. 2021; 10: (10). https://doi.org/10.3390/antiox10101626.

81. Ye L, Hu ZP, Du GY, Zhang JZ, Dong QJ, Fu FH, Tian JW. Antidepressant-like effects of the extract from Cimicifuga foetida L. J Ethnopharmacol. 2012; 144: (3), 683-691. https://doi.org/10.1016/j.jep.2012.10.013.

82. Gerin F, Erman H, Erboga M, Sener U, Yilmaz A, Seyhan H, Gurel A. The Effects of Ferulic Acid Against Oxidative Stress and Inflammation in Formaldehyde-Induced Hepatotoxicity. Inflammation. 2016; 39: (4), 1377-86. https://doi.org/10.1007/s10753-016-0369-4.

83. Jia H, Wang XY, Liu WW, Qin XC, Hu B, Ma Q, Lv CN, Lu JC. Cimicifuga dahurica extract inhibits the proliferation, migration and invasion of breast cancer cells MDA-MB-231 and MCF-7 in vitro and in vivo. J Ethnopharmacol. 2021; 277. https://doi.org/10.1016/j.jep.2021.114057.

84. Yang ZC, Ma J. Actein enhances TRAIL effects on suppressing gastric cancer progression by activating p53/Caspase-3 signaling. Biochem Bioph Res Co. 2018; 497: (4), 1177-1183. https://doi.org/10.1016/j.bbrc.2016.11.162.

85. Jia H, Liu M, Wang X, Jiang Q, Wang S, Santhanam RK, Lv C, Zhao Q, Lu J. Cimigenoside functions as a novel γ-secretase inhibitor and inhibits the proliferation or metastasis of human breast cancer cells by γ-secretase/Notch axis. Pharmacol Res. 2021; 169, 105686. https://doi.org/10.1016/j.phrs.2021.105686.

86. Wang MS, Chen L, Xiong YQ, Xu J, Wang JP, Meng ZL. Iron oxide magnetic nanoparticles combined with actein suppress non-small-cell lung cancer growth in a p53-dependent manner. Int J Nanomed. 2017; 12, 7627-+. https://doi.org/10.2147/ijn.S127549.

87. Zhao CG, Zhang ZL, Dai XH, Wang JH, Liu H, Ma HW. Actein Antagonizes Oral Squamous Cell Carcinoma Proliferation through Activating FoxO1. Pharmacology. 2021; 106: (9-10), 551-563. https://doi.org/10.1159/000515601.

88. Long Z, Feng G, Zhao N, Wu L, Zhu H. Isoferulic acid inhibits human leukemia cell growth through induction of G2/M‑phase arrest and inhibition of Akt/mTOR signaling. Mol Med Rep. 2020; 21: (3), 1035-1042. https://doi.org/10.3892/mmr.2020.10926.

89. Wang QQ, Yang H, Yang WJ, Jiang TS. Cimigenoside Affects Cell Viability, Apoptosis and Metastasis of A549 Cells via the NF-kappa B Pathway. Folia Biologica-Krakow. 2022; 70: (2), 85-91. https://doi.org/10.3409/fb_70-2.10.

90. Sun H, Huang M, Yao N, Hu J, Li Y, Chen L, Hu N, Ye W, Chi-Shing Tai W, Zhang D, Chen S. The cycloartane triterpenoid ADCX impairs autophagic degradation through Akt overactivation and promotes apoptotic cell death in multidrug-resistant HepG2/ADM cells. Biochem Pharmacol. 2017; 146, 87-100. https://doi.org/10.1016/j.bcp.2017.10.012.

91. Dai XL, Liu J, Nian Y, Qiu MH, Luo Y, Zhang JH. A novel cycloartane triterpenoid from Cimicifuga induces apoptotic and autophagic cell death in human colon cancer HT-29 cells. Oncol Rep. 2017; 37: (4), 2079-2086. https://doi.org/10.3892/or.2017.5444.

92. Ma YP, Cong WJ, Huang H, Sun L, Mai AH, Boonen K, Maryam W, De Borggraeve W, Luo GA, Liu QF, Schoofs L, Van Kuppeveld F, Neyts J, Mirabelli C, Luyten W. Identification of fukinolic acid from Cimicifuga heracleifolia and its derivatives as novel antiviral compounds against enterovirus A71 infection. International journal of antimicrobial agents. 2019; 53: (2), 128-136. https://doi.org/10.1016/j.ijantimicag.2018.07.014.

93. Wang KC, Chang JS, Chiang LC, Lin CC. Cimicifuga foetida L. inhibited human respiratory syncytial virus in HEp-2 and A549 cell lines. Am J Chinese Med. 2012; 40: (1), 151-162. https://doi.org/10.1142/s0192415x12500127.

94. Wang KC, Chang JS, Lin LT, Chiang LC, Lin CC. Antiviral effect of cimicifugin from Cimicifuga foetida against human respiratory syncytial virus. Am J Chinese Med. 2012; 40: (5), 1033-1045. https://doi.org/10.1142/s0192415x12500760.

95. Wang YP, Ma D, Cheng XT, Zhang SJ, Xue W, Deng Y, Wang YF, Sun AJ. Comparison of Cimicifuga foetida extract and different hormone therapies regarding in causing breast pain in early postmenopausal women. Gynecol Endocrinol. 2019; 35: (2), 160-164. https://doi.org/10.1080/09513590.2018.1505845.

96. Yuan J, Shi Q, Chen J, Lu J, Wang L, Qiu M, Liu J. Effects of 23-epi-26-deoxyactein on adipogenesis in 3T3-L1 preadipocytes and diet-induced obesity in C57BL/6 mice. Phytomedicine. 2020; 76, 153264. https://doi.org/10.1016/j.phymed.2020.153264.

97. Lee JE, Kim BB, Ko Y, Jeong SH, Park JB. Effects of Cimicifugae Rhizoma on the osteogenic and adipogenic differentiation of stem cells. Exp Ther Med. 2017; 13: (2), 443-448. https://doi.org/10.3892/etm.2016.4010.

98. Kim JH, Thao NP, Han YK, Lee YS, Luyen BTT, Oanh HV, Kim YH, Yang SY. The insight of in vitro and in silico studies on cholinesterase inhibitors from the roots of Cimicifuga dahurica (Turcz.) Maxim. J Enzyme Inhib Med Chem. 2018; 33: (1), 1174-1180. https://doi.org/10.1080/14756366.2018.1491847.

99. Lee SB, Yang SY, Thao NP, Seo DG, Kim S, Ma CT, Park SY, Kim YH, Yang HO. Protective Effects of Compounds from Cimicifuga dahurica against Amyloid Beta Production in Vitro and Scopolamine-Induced Memory Impairment in Vivo. J Nat Prod. 2020; 83: (2), 223-230. https://doi.org/10.1021/acs.jnatprod.9b00543.

100. Qin R, Zhao Y, Zhao Y, Zhou W, Lv C, Lu J. Polyphenolic compounds with antioxidant potential and neuro-protective effect from Cimicifuga dahurica (Turcz.) Maxim. Fitoterapia. 2016; 115, 52-56. https://doi.org/10.1016/j.fitote.2016.09.016.

101. Miao LY, Chu TTH, Li P, Jiang Y, Li HJ. Cimicifuga heracleifolia is therapeutically similar to black cohosh in relieving menopausal symptoms: evidence from pharmacological and metabolomics studies. Chinese Journal of Natural Medicines. 2019; 17: (6), 435-445. https://doi.org/10.1016/s1875-5364(19)30051-2.

102. Hu X, Qi C, Feng F, Wang Y, Di T, Meng Y, Wang Y, Zhao N, Zhang X, Li P, Zhao J. Combining network pharmacology, RNA-seq, and metabolomics strategies to reveal the mechanism of Cimicifugae Rhizoma - Smilax glabra Roxb herb pair for the treatment of psoriasis. Phytomedicine. 2022; 105, 154384. https://doi.org/10.1016/j.phymed.2022.154384.
